# Mathematical modeling of tumor therapy with oncolytic viruses: effects of parametric heterogeneity on cell dynamics

**DOI:** 10.1186/1745-6150-1-30

**Published:** 2006-10-03

**Authors:** Georgy P Karev, Artem S Novozhilov, Eugene V Koonin

**Affiliations:** 1 National Center for Biotechnology Information, National Library of Medicine, National Institutes of Health, Bethesda, MD 20894, USA

## Abstract

**Background:**

One of the mechanisms that ensure cancer robustness is tumor heterogeneity, and its effects on tumor cells dynamics have to be taken into account when studying cancer progression. There is no unifying theoretical framework in mathematical modeling of carcinogenesis that would account for parametric heterogeneity.

**Results:**

Here we formulate a modeling approach that naturally takes stock of inherent cancer cell heterogeneity and illustrate it with a model of interaction between a tumor and an oncolytic virus. We show that several phenomena that are absent in homogeneous models, such as cancer recurrence, tumor dormancy, and others, appear in heterogeneous setting. We also demonstrate that, within the applied modeling framework, to overcome the adverse effect of tumor cell heterogeneity on the outcome of cancer treatment, a heterogeneous population of an oncolytic virus must be used. Heterogeneity in parameters of the model, such as tumor cell susceptibility to virus infection and the ability of an oncolytic virus to infect tumor cells, can lead to complex, irregular evolution of the tumor. Thus, quasi-chaotic behavior of the tumor-virus system can be caused not only by random perturbations but also by the heterogeneity of the tumor and the virus.

**Conclusion:**

The modeling approach described here reveals the importance of tumor cell and virus heterogeneity for the outcome of cancer therapy. It should be straightforward to apply these techniques to mathematical modeling of other types of anticancer therapy.

**Reviewers:**

Leonid Hanin (nominated by Arcady Mushegian), Natalia Komarova (nominated by Orly Alter), and David Krakauer.

## Open peer review

Reviewed by Leonid Hanin (nominated by Arcady Mushegian), Natalia Komarova (nominated by Orly Alter), and David Krakauer.

For the full reviews, please go to the Reviewers' comments section.

## Background

Cancers are extremely complex systems that possess many features of robustness, i.e., they are systems that tend to maintain stable functioning despite various perturbations [1,2]. The main two mechanisms that enable cancer robustness are functional redundancy, that comes from inherent heterogeneity of tumor cells, and feedback-control systems that facilitate survival of a tumor under adverse conditions, e.g., caused by anticancer drugs [3-6]. Many approaches to anticancer treatment have had limited success due to cancer robustness. An important challenge is to identify fragilities of cancers as robust biological systems and develop treatment strategies that take advantage of these weak links. Thus, a better understanding of the mechanisms that yield cancer robustness, particularly, functional redundancy, is vital.

Redundancy in cancers occurs at two levels. First, multiple copies of identical cells increase the likelihood of restoration of a tumor after treatment. Second, functional redundancy can be mediated by functionally equivalent but heterogeneous components (known as heterogeneous redundancy). Heterogeneous redundancy is thought to be central to tumor robustness. Although heterogeneity is a well-recognized characteristic of tumors that can lead to drug resistance, the current body of experimental and clinical data that relate to dynamic changes in intratumoral heterogeneity during progression, as well as to responses to various therapeutic strategies, is insufficient [2].

The term 'tumor heterogeneity' means the existence of distinct subpopulations of tumor cells with specific characteristics within a single neoplasm [7,8]; in particular, surviving subpopulations of cells with metastatic potential after anticancer therapy can lead to tumor recurrence [9-11]. Tumor heterogeneity is a well documented phenomenon [3] as demonstrated by extensive cytogenetic analysis [12,13]. Furthermore, many tumors are characterized by heterogeneous, often non-random, spatio-temporal distribution of genetically heterogeneous tumor cells [4]. Distinct subpopulations of cells have been isolated from experimental and human neoplasms of every major histological type and location. These subpopulations are heterogeneous for many characteristics, such as morphology, growth rate, metastatic potential, karyotype, antigenicity, immunogenicity, biochemical properties, sensitivity to chemotherapeutic agents and radiation, etc. Genetic heterogeneity is, arguably, a major cause of acquired drug resistance of tumors [14,15].

The dynamics of host-tumor system, which entails co-evolution under the selective pressure that is imposed by host environments, including antitumor drugs, is highly complex and nonlinear. Thus, to precisely define the conditions for successful therapy, mathematical models are needed. Extensive efforts have been dedicated over many years to mathematical modeling of cancer development and anticancer therapy. Stochastic models that take into account random mutations and cell proliferation proved to be useful in the context of epidemiology and statistical data [16], and for modeling cancer initiation and progression in terms of somatic evolution [17]. Deterministic models of tumor growth have proved valuable as well. Many of these have addressed avascular and vascular tumor growth taking advantage of methods borrowed from physics [18] but some use models from population biology to treat a tumor as a dynamic society of interacting cells [19-21]. A variety of mathematical approaches contribute to modeling cancer progression from different standpoints and take stock of various factors affecting tumor growth [[22] and references therein, [23]]. Combined analysis of tumor growth and anticancer therapies within the framework of mathematical modeling also produced a number of significant results [24,25].

Inasmuch as tumor heterogeneity is one of the crucial factors in determining possible outcomes of anticancer treatment, it has to be incorporated and investigated in mathematical models. Although heterogeneity of tumor cell populations has been widely studied, there is no general approach that would provide a comprehensive description of intrinsic heterogeneity of tumors and would be amenable to qualitative and quantitative mathematical analysis. The existing mathematical models of anticancer therapy either do not include the effects of heterogeneity [e.g., [26,27]], or consider finite, usually, small, number of subpopulations, e.g., sensitive and resistant cells or proliferating and quiescent cells [e.g., [28,29]]. Other models take into account only spatial heterogeneity and do not address the important subject of genetic heterogeneity [e.g., [30-32]], or use individual-based approach and simulation techniques and thus are, practically, not amenable to theoretical mathematical analysis [e.g., [33-35]].

The main goal of the present paper is to introduce a novel approach to model heterogeneity into the field of cancer modeling. We show that heterogeneous models, although still oversimplified, reflect qualitatively new phenomena, some of which are observed in experiments and clinical trials. We present an effective method to analyze these models and examine the predictions that such heterogeneous models can yield.

We illustrate our approach by addressing a complex process that involves both virus-cell interaction and tumor growth, namely, the interaction of the so-called oncolytic viruses with tumors. Oncolytic viruses are viruses that specifically infect and kill cancer cells but do not affect normal cells [36-39]. Many types of oncolytic viruses have been studied as candidate therapeutic agents, including adenoviruses, herpesviruses, poxviruses, reoviruses, paramyxoviruses, and retroviruses [37,39]. Probably, the best-characterized oncolytic virus, that has drawn much attention, is ONYX-015, an attenuated adenovirus that selectively infects tumor cells with a defect in the *p53 *gene [38,40]. This virus has been shown to possess substantial antitumor activity and has proven relatively effective at reducing or eliminating tumors in clinical trials [41-43]. Although safety and efficacy remain major concerns, several other oncolytic viruses acting on different principles, including tumor-specific transcription of the viral genome, have been developed, and some of these viruses have entered or are about to enter clinical trials [37,44-47]. Recently, synergistic use of immune therapy and oncolytic viruses has shown particular promise in cancer treatment [48].

The oncolytic effect can result from at least three distinct modes of virus-host interaction [37,39]. The first mode involves repeated cycles of viral replication in the tumor cells leading to cell death and, consequently, to tumor reduction and, potentially, elimination. The second mode involves low-level virus reproduction that, however, results in the production of a cytotoxic protein that causes cell damage. The third mode consists in induction of antitumor immunity by virus infection of cancer cells. Cancer cells possess weak antigens for host immune sensitization. Virus infection causes inflammation and lymphocyte penetration into the tumor, with the virus antigens eliciting increased sensitivity to tumor necrosis factor-mediated killing.

Although the indirect modes of virus cancer therapy based on production of cytotoxic proteins or antitumor immunity might be promising, direct lysis of tumor cells by an oncolytic virus is the current mainstream strategy. Experiments on human tumor xenografts in nude mice have shown that the effect of oncolytic virus infection on tumors can range from no apparent effect, to reduction and stabilization of the tumor load (i.e., the overall size of a tumor), to elimination of the tumor [49]. Complete regression of tumors has been reported also in some patients treated with oncolytic viruses as part of clinical trials [50]. In a previous study [51], we presented a conceptual mathematical model of tumor cells-virus interaction which, depending on system parameter values, exhibits various behaviors including deterministic elimination of the cancer cells. Here, we further examine this model in conjunction with different possible heterogeneities of tumor cells, such as different susceptibility to infection, different death rates of infected cells, and others.

## Results

### Homogeneous mathematical models (phase-parameter portrait)

In this section, we briefly present the main results of the analysis of a simple conceptual model of virus-tumor interaction [51]; these will be important for the further analysis described in the present work. The model allows for two populations of cells: uninfected tumor cells and infected tumor cells and is an extension of the previous work of Wodarz [52] to include an alternative non-linear functional response for infection rate.

The model, which considers two types of cells growing in the logistic fashion, has the following form:

dXdt=r1X(1−X+YK)−bXYX+Y,dYdt=r2Y(1−X+YK)+bXYX+Y−aY,     (1)
 MathType@MTEF@5@5@+=feaafiart1ev1aaatCvAUfKttLearuWrP9MDH5MBPbIqV92AaeXatLxBI9gBaebbnrfifHhDYfgasaacH8akY=wiFfYdH8Gipec8Eeeu0xXdbba9frFj0=OqFfea0dXdd9vqai=hGuQ8kuc9pgc9s8qqaq=dirpe0xb9q8qiLsFr0=vr0=vr0dc8meaabaqaciaacaGaaeqabaqabeGadaaakeaafaqaaeGabaaabaWaaSaaaeaacqWGKbazcqWGybawaeaacqWGKbazcqWG0baDaaGaeyypa0JaemOCai3aaSbaaSqaaiabigdaXaqabaGccqWGybawdaqadiqaaiabigdaXiabgkHiTmaalaaabaGaemiwaGLaey4kaSIaemywaKfabaGaem4saSeaaaGaayjkaiaawMcaaiabgkHiTmaalaaabaGaemOyaiMaemiwaGLaemywaKfabaGaemiwaGLaey4kaSIaemywaKfaaiabcYcaSaqaamaalaaabaGaeeizaqMaemywaKfabaGaeeizaqMaemiDaqhaaiabg2da9iabdkhaYnaaBaaaleaacqaIYaGmaeqaaOGaemywaK1aaeWaceaacqaIXaqmcqGHsisldaWcaaqaaiabdIfayjabgUcaRiabdMfazbqaaiabdUealbaaaiaawIcacaGLPaaacqGHRaWkdaWcaaqaaiabdkgaIjabdIfayjabdMfazbqaaiabdIfayjabgUcaRiabdMfazbaacqGHsislcqWGHbqycqWGzbqwcqGGSaalaaGaaCzcaiaaxMaadaqadiqaaiabigdaXaGaayjkaiaawMcaaaaa@69FF@

where *X *is the size of the uninfected cell population; *Y *is the size of the infected cell population; *r*_1 _and *r*_2 _are the maximum per capita growth rates of uninfected and infected cells, respectively; *K *is the carrying capacity; *b *is the transmission coefficient (this parameter may also include the replication rate of the virus); and *a *is the rate of infected cell killing by the virus (cytotoxicity). All the parameters of the model are supposed to be non-negative. Model (1) is subject to initial conditions *X*(0) = *X*_0 _> 0 and *Y*(0) = *Y*_0 _> 0. The concentration of viral particles is not explicitly included; it is assumed that virus abundance is proportional to infected cell abundance [53].

Rescaling model (1) by letting X^* ^(t^*^) = *X*(*t*)/*K*, Y^* ^(t^*^) = *Y *(*t*)/*K*, t^* ^= *r*_1_*t *leads to the system

dX∗dt∗=X∗(1−(X∗+Y∗))−βX∗Y∗X*+Y∗,dY∗dt∗=γY∗(1−(X∗+Y∗))+βX∗Y∗X∗+Y∗−δY∗,     (2)
 MathType@MTEF@5@5@+=feaafiart1ev1aaatCvAUfKttLearuWrP9MDH5MBPbIqV92AaeXatLxBI9gBaebbnrfifHhDYfgasaacH8akY=wiFfYdH8Gipec8Eeeu0xXdbba9frFj0=OqFfea0dXdd9vqai=hGuQ8kuc9pgc9s8qqaq=dirpe0xb9q8qiLsFr0=vr0=vr0dc8meaabaqaciaacaGaaeqabaqabeGadaaakeaafaqaaeGabaaabaWaaSaaaeaacqWGKbazcqWGybawdaahaaWcbeqaaiabgEHiQaaaaOqaaiabdsgaKjabdsha0naaCaaaleqabaGaey4fIOcaaaaakiabg2da9iabdIfaynaaCaaaleqabaGaey4fIOcaaOGaeiikaGIaeGymaeJaeyOeI0IaeiikaGIaemiwaG1aaWbaaSqabeaacqGHxiIkaaGccqGHRaWkcqWGzbqwdaahaaWcbeqaaiabgEHiQaaakiabcMcaPiabcMcaPiabgkHiTmaalaaabaacciGae8NSdiMaemiwaG1aaWbaaSqabeaacqGHxiIkaaGccqWGzbqwdaahaaWcbeqaaiabgEHiQaaaaOqaaiabdIfaynaaCaaaleqabaGaeiOkaOcaaOGaey4kaSIaemywaK1aaWbaaSqabeaacqGHxiIkaaaaaOGaeiilaWcabaWaaSaaaeaacqWGKbazcqWGzbqwdaahaaWcbeqaaiabgEHiQaaaaOqaaiabdsgaKjabdsha0naaCaaaleqabaGaey4fIOcaaaaakiabg2da9iab=n7aNjabdMfaznaaCaaaleqabaGaey4fIOcaaOGaeiikaGIaeGymaeJaeyOeI0IaeiikaGIaemiwaG1aaWbaaSqabeaacqGHxiIkaaGccqGHRaWkcqWGzbqwdaahaaWcbeqaaiabgEHiQaaakiabcMcaPiabcMcaPiabgUcaRmaalaaabaGae8NSdiMaemiwaG1aaWbaaSqabeaacqGHxiIkaaGccqWGzbqwdaahaaWcbeqaaiabgEHiQaaaaOqaaiabdIfaynaaCaaaleqabaGaey4fIOcaaOGaey4kaSIaemywaK1aaWbaaSqabeaacqGHxiIkaaaaaOGaeyOeI0Iae8hTdqMaemywaK1aaWbaaSqabeaacqGHxiIkaaGccqGGSaalaaGaaCzcaiaaxMaadaqadiqaaiabikdaYaGaayjkaiaawMcaaaaa@7E91@

where *β *= *b*/*r*_1_, *γ *= *r*_2_/*r*_1_, and *δ *= *a*/*r*_1_. In the following analysis, we suppress the asterisks to simplify the notations, but it should be clear that we use non-dimensional parameters and scaled sizes of cell populations, so that *X *+ *Y *≤ 1 for any *t*.

**Figure 1:**
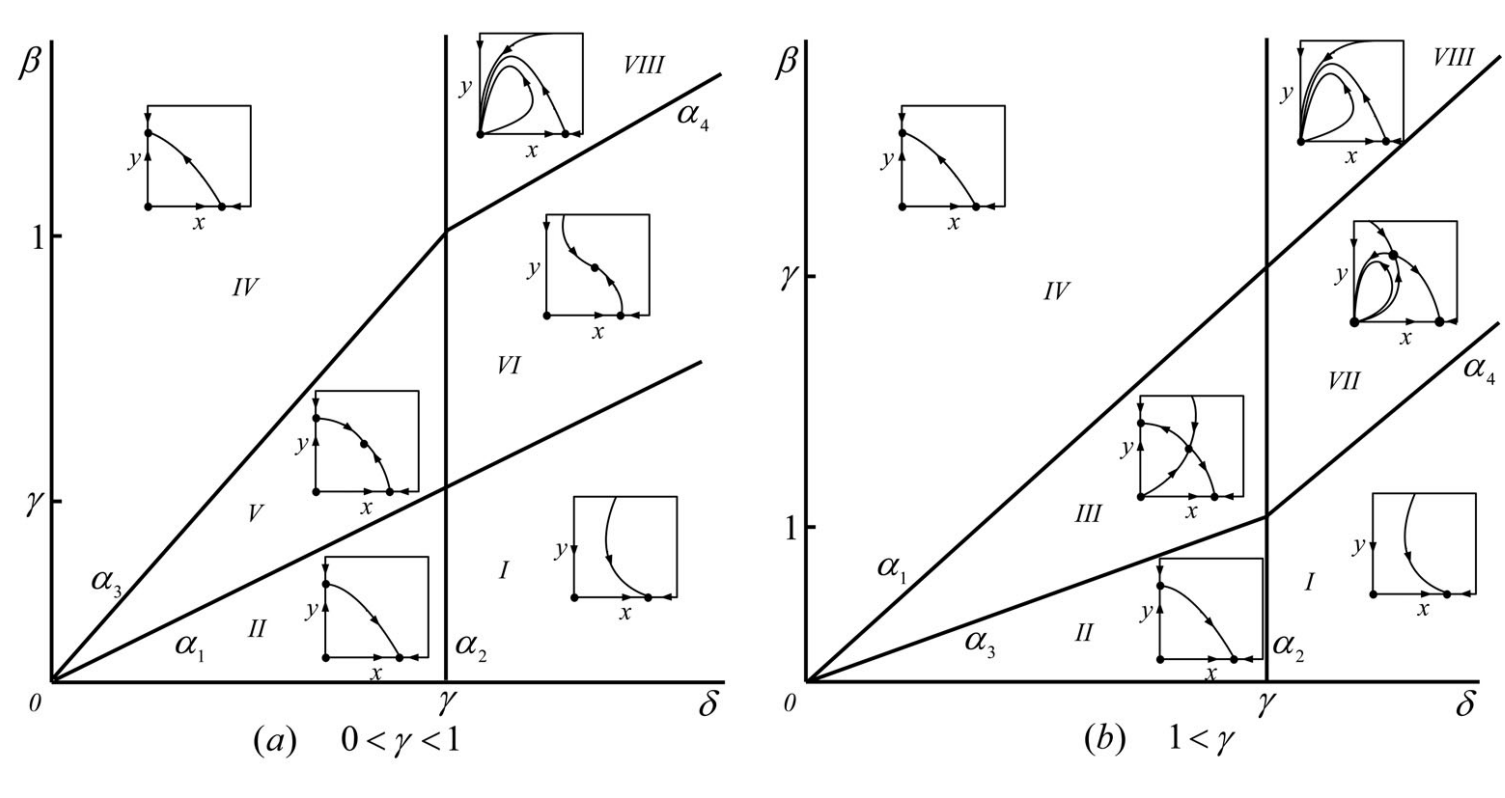
Phase-parameter portrait of system (2) given as a cut of the positive parameter space (*γ*, *β*, *δ*) for an arbitrary fixed value of 0 <*γ *< 1 (a) and 1 <*γ *(b). The boundaries of domains are α_1 _= {(*δ*, *β*, *γ*): *δ *- *β *= 0},α_2 _= {(*δ*, *β*, *γ*): *γ *-*δ *= 0}, α_3 _= {(*δ*, *β*, *γ*): *γβ *-*δ *= 0}, and α_4 _= {(*δ*, *β*, *γ*): *γ *- *δ *- 1 + *β *= 0}.

The complete phase-parameter portrait of system (2) is shown in Fig. 1.

The model (2) exhibits all possible outcomes of oncolytic virus infection, i.e., no effect on the tumor (domains *I *and *II *in Fig. 1), stabilization or reduction of the tumor load (domains *IV *and *V*), and complete elimination of the tumor (domain *VIII*). Moreover there are two domains (domains *III *and *VII*) where the final outcome crucially depends on the initial conditions and can result either in failure of virus therapy or in stabilization (domain *III*) and elimination (domain *VII*) of the tumor.

### Heterogeneous model

#### General consideration

The model (1) consists of two coupled, deterministic differential equations allowing for cell reproduction and death, and cell infection. This model is one of the conceptual mathematical models of tumor growth that treat a tumor as a dynamic society of interacting cells (e.g., [19,20,22]). Most population models suppose that all individuals have identical attributes, in particular, identical rates of growth, death and birth. This assumption simplifies computation, albeit at the cost of realism.

There are different ways to model heterogeneity. If it is assumed that heterogeneity of population is mediated by a structured variable (such as explicit space or age), we have to deal with partial differential equations (PDEs) (for application of space explicit models to tumor-virus interaction, see [54] and references therein; cell age is modeled, e.g., in [55]). By contrast, here, we explore heterogeneity of parameters (such as birth and death rates, and infection rate), i.e., we consider parameters as inherent and invariable properties of individuals, whereas parameter values can vary between individuals; any changes of mean, variance and other characteristics of the parameter distribution with time are caused only by variation of the population structure due to the system dynamics; the distribution of parameter values changes with time because different subpopulations evolve with different, non-constant rates. This type of heterogeneity has been designated 'parametric' by Dushoff [56].

The most common method to take account of parametric heterogeneity is to divide a population into groups [28,57,58]. An important disadvantage of this approach is that heterogeneity within a group cannot be incorporated and the number of groups usually should be small because, otherwise, the dimension of the model is too large for qualitative analysis. Another approach is to consider the population as having a continuous distribution of parameters [59-62]. In the latter case, one usually has to deal with infinite-dimensional dynamical systems but, in a wide range of specific cases, there is an efficient analytical theory that allows one to reduce an initial distributed system to a system of ordinary differential equations (ODEs) [61,62] (see also Mathematical Appendix (hereinafter MA) [see Additional file 1]). Here, we adopted this approach and emphasize that the applied reduction permits us to follow not only the total sizes of the populations, but also the time-dependent dynamics of the distributions, i.e., no information is lost when we switch from infinite-dimensional dynamical system to system of ODEs. It should be further emphasized that this reduction is not an approximation but results in an equivalent model, in contrast to the approaches commonly used to solve similar systems numerically [59,63,64]. Moreover, this approach can be used for arbitrary distributions, both continuous and discrete, with the mathematical formalism remaining the same.

The starting point of our analysis is the model (2) which is a non-dimensional version of the initial model (1). As a matter of fact, by assuming that one or more parameters are distributed through the populations of cells, we have to deal with the initial system (1) with dimensional parameters. However, the two parameters that are of particular interest are transmission coefficient *b *and cytotoxicity *a*, and in the following we assume that these parameters (or either of them) are distributed, whereas the net growth rates and carrying capacity are supposed to be the same for all tumor cells. Since non-dimensional parameters *β *and *δ *differ from *b *and *a *only by linear scaling, the system with distributed non-dimensional parameters *β *and *δ *can be analyzed without loss of generality.

#### Distributed susceptibility

Let us assume that transmission coefficient *β *is distributed through the population of uninfected tumor cells. Denote B the set of possible values of *β *and *x*(*t*, *β*) the density of uninfected cells that have a given value of *β *at moment *t *(i.e., for any subset  B˜
 MathType@MTEF@5@5@+=feaafiart1ev1aaatCvAUfKttLearuWrP9MDH5MBPbIqV92AaeXatLxBI9gBaebbnrfifHhDYfgasaacH8akY=wiFfYdH8Gipec8Eeeu0xXdbba9frFj0=OqFfea0dXdd9vqai=hGuQ8kuc9pgc9s8qqaq=dirpe0xb9q8qiLsFr0=vr0=vr0dc8meaabaqaciaacaGaaeqabaqabeGadaaakeaacuqGcbGqgaacaaaa@2DC6@ ⊆ B, the part of the population with trait values belonging to  B˜
 MathType@MTEF@5@5@+=feaafiart1ev1aaatCvAUfKttLearuWrP9MDH5MBPbIqV92AaeXatLxBI9gBaebbnrfifHhDYfgasaacH8akY=wiFfYdH8Gipec8Eeeu0xXdbba9frFj0=OqFfea0dXdd9vqai=hGuQ8kuc9pgc9s8qqaq=dirpe0xb9q8qiLsFr0=vr0=vr0dc8meaabaqaciaacaGaaeqabaqabeGadaaakeaacuqGcbGqgaacaaaa@2DC6@ is given by ∫
													
       B˜x(t,β)dβ
 MathType@MTEF@5@5@+=feaafiart1ev1aaatCvAUfKttLearuWrP9MDH5MBPbIqV92AaeXatLxBI9gBaebbnrfifHhDYfgasaacH8akY=wiFfYdH8Gipec8Eeeu0xXdbba9frFj0=OqFfea0dXdd9vqai=hGuQ8kuc9pgc9s8qqaq=dirpe0xb9q8qiLsFr0=vr0=vr0dc8meaabaqaciaacaGaaeqabaqabeGadaaakeaadaWdraqaaiabdIha4jabcIcaOiabdsha0jabcYcaSGGaciab=j7aIjabcMcaPiabdsgaKjab=j7aIbWcbaGafeOqaiKbaGaaaeqaniabgUIiYdaaaa@39E1@, and the total size of the population, *X *(*t*), is given by ∫Bx(t,β)dβ
 MathType@MTEF@5@5@+=feaafiart1ev1aaatCvAUfKttLearuWrP9MDH5MBPbIqV92AaeXatLxBI9gBaebbnrfifHhDYfgasaacH8akY=wiFfYdH8Gipec8Eeeu0xXdbba9frFj0=OqFfea0dXdd9vqai=hGuQ8kuc9pgc9s8qqaq=dirpe0xb9q8qiLsFr0=vr0=vr0dc8meaabaqaciaacaGaaeqabaqabeGadaaakeaadaWdraqaaiabdIha4jabcIcaOiabdsha0jabcYcaSGGaciab=j7aIjabcMcaPiabdsgaKjab=j7aIbWcbaGaeeOqaieabeqdcqGHRiI8aaaa@39D2@).

The number of uninfected cells with a given value of *β *that become infected per time unit is given by *ρx*(*t*, *β*), where *ρ *= *ρ*(*β*, *X*, *Y*) is the rate of infection. We assume that the rate of infection depends only on the value of *β *and the total sizes of the infected and uninfected cell subpopulations. This assumption reflects the fact that different cells can have different susceptibilities to virus infection, i.e., some of them can be infected with a relatively high probability whereas others are infected with a low or even arbitrary, close to zero probability; the latter case corresponds to the situation when a particular subpopulation of tumor cells can hardly be infected by the virus.

Using the nonlinear transmission function in (2) we obtain that the change in subpopulation of uninfected cells with parameter value *β *is given by *βx*(*t*, *β*)*Y*(*t*)/(*X*(*t*) + *Y*(*t*)). We also need to specify the law of growth of tumor cells with a fixed value of *β*. Let us assume that uninfected cells with a particular value of *β *beget daughter cells with the same parameter value. For the net growth rate, we use the logistic law that depends on the total population sizes, *X *and *Y*. We do not consider heterogeneity in the infected cell population, and, hence, the total amount of newly infected cells per time unit is given by *Y*(*t*) ∫Bβx(t,β)dβ
 MathType@MTEF@5@5@+=feaafiart1ev1aaatCvAUfKttLearuWrP9MDH5MBPbIqV92AaeXatLxBI9gBaebbnrfifHhDYfgasaacH8akY=wiFfYdH8Gipec8Eeeu0xXdbba9frFj0=OqFfea0dXdd9vqai=hGuQ8kuc9pgc9s8qqaq=dirpe0xb9q8qiLsFr0=vr0=vr0dc8meaabaqaciaacaGaaeqabaqabeGadaaakeaadaWdraqaaGGaciab=j7aIjabdIha4jabcIcaOiabdsha0jabcYcaSiab=j7aIjabcMcaPiabdsgaKjab=j7aIbWcbaGaeeOqaieabeqdcqGHRiI8aaaa@3B6E@/*X*(*t*) + *Y*(*t*)), which should be taken into account when writing down the equation for the change in the infected cell population. By specifying initial conditions for *x*(*t*, *β*) and *Y*(*t*), we obtain the model in the form

∂x(t,β)∂t=x(t,β)[1−(X(t)+Y(t))]−βx(t,β)Y(t)X(t)+Y(t),dY(t)dt=γY(t)[1−(X(t)+Y(t))]+Eβ(t)X(t)Y(t)X(t)+Y(t)−δY(t),     (3)
 MathType@MTEF@5@5@+=feaafiart1ev1aaatCvAUfKttLearuWrP9MDH5MBPbIqV92AaeXatLxBI9gBaebbnrfifHhDYfgasaacH8akY=wiFfYdH8Gipec8Eeeu0xXdbba9frFj0=OqFfea0dXdd9vqai=hGuQ8kuc9pgc9s8qqaq=dirpe0xb9q8qiLsFr0=vr0=vr0dc8meaabaqaciaacaGaaeqabaqabeGadaaakeaafaqaaeGabaaabaWaaSaaaeaacqGHciITcqWG4baEcqGGOaakcqWG0baDcqGGSaaliiGacqWFYoGycqGGPaqkaeaacqGHciITcqWG0baDaaGaeyypa0JaemiEaGNaeiikaGIaemiDaqNaeiilaWIae8NSdiMaeiykaKIaei4waSLaeGymaeJaeyOeI0IaeiikaGIaemiwaGLaeiikaGIaemiDaqNaeiykaKIaey4kaSIaemywaKLaeiikaGIaemiDaqNaeiykaKIaeiykaKIaeiyxa0LaeyOeI0YaaSaaaeaacqWFYoGycqWG4baEcqGGOaakcqWG0baDcqGGSaalcqWFYoGycqGGPaqkcqWGzbqwcqGGOaakcqWG0baDcqGGPaqkaeaacqWGybawcqGGOaakcqWG0baDcqGGPaqkcqGHRaWkcqWGzbqwcqGGOaakcqWG0baDcqGGPaqkaaGaeiilaWcabaWaaSaaaeaacqWGKbazcqWGzbqwcqGGOaakcqWG0baDcqGGPaqkaeaacqWGKbazcqWG0baDaaGaeyypa0Jae83SdCMaemywaKLaeiikaGIaemiDaqNaeiykaKIaei4waSLaeGymaeJaeyOeI0IaeiikaGIaemiwaGLaeiikaGIaemiDaqNaeiykaKIaey4kaSIaemywaKLaeiikaGIaemiDaqNaeiykaKIaeiykaKIaeiyxa0Laey4kaSYaaSaaaeaacqWGfbqrdaWgaaWcbaGae8NSdigabeaakiabcIcaOiabdsha0jabcMcaPiabdIfayjabcIcaOiabdsha0jabcMcaPiabdMfazjabcIcaOiabdsha0jabcMcaPaqaaiabdIfayjabcIcaOiabdsha0jabcMcaPiabgUcaRiabdMfazjabcIcaOiabdsha0jabcMcaPaaacqGHsislcqWF0oazcqWGzbqwcqGGOaakcqWG0baDcqGGPaqkcqGGSaalaaGaaCzcaiaaxMaadaqadiqaaiabiodaZaGaayjkaiaawMcaaaaa@AC8D@

where the following notation was used: *E*_*β *_(*t*) = (∫Bβx(t,β)dβ
 MathType@MTEF@5@5@+=feaafiart1ev1aaatCvAUfKttLearuWrP9MDH5MBPbIqV92AaeXatLxBI9gBaebbnrfifHhDYfgasaacH8akY=wiFfYdH8Gipec8Eeeu0xXdbba9frFj0=OqFfea0dXdd9vqai=hGuQ8kuc9pgc9s8qqaq=dirpe0xb9q8qiLsFr0=vr0=vr0dc8meaabaqaciaacaGaaeqabaqabeGadaaakeaadaWdraqaaGGaciab=j7aIjabdIha4jabcIcaOiabdsha0jabcYcaSiab=j7aIjabcMcaPiabdsgaKjab=j7aIbWcbaGaeeOqaieabeqdcqGHRiI8aaaa@3B6E@)/*X*(*t*).

The initial conditions are

*x*(0, *β*) = *x*_0 _(*β*) = *X*_0_*p*(0, *β*), *Y*(0) = *Y*_0_.       (4)

Here *x*_0_(*β*) is the initial distribution of *β *in the population of uninfected cells, *X*_0 _is the total number of uninfected cells at the initial moment, and *p*(0, *β*) is the probability density function (pdf) of the initial distribution of. *β*. Note that *E*_*β *_(*t*) can be rewritten in the form *E*_*β *_(*t*) = ∫Bβp(t,β)dβ
 MathType@MTEF@5@5@+=feaafiart1ev1aaatCvAUfKttLearuWrP9MDH5MBPbIqV92AaeXatLxBI9gBaebbnrfifHhDYfgasaacH8akY=wiFfYdH8Gipec8Eeeu0xXdbba9frFj0=OqFfea0dXdd9vqai=hGuQ8kuc9pgc9s8qqaq=dirpe0xb9q8qiLsFr0=vr0=vr0dc8meaabaqaciaacaGaaeqabaqabeGadaaakeaadaWdraqaaGGaciab=j7aIjabdchaWjabcIcaOiabdsha0jabcYcaSiab=j7aIjabcMcaPiabdsgaKjab=j7aIbWcbaGaeeOqaieabeqdcqGHRiI8aaaa@3B5E@, where *p*(*t*, *β*) is the pdf of the distribution of *β *at time *t*, and, thus, *E*_*β *_(*t*) is the mean value of *β *at moment *t*.

Integrating the first equation in (3) over *β *we obtain the system

dXdt=X(1−(x+Y))−Eβ(t)XYX+Y,dYdt=γY(1−(X+Y))+Eβ(t)XYX+Y−δY,     (5)
 MathType@MTEF@5@5@+=feaafiart1ev1aaatCvAUfKttLearuWrP9MDH5MBPbIqV92AaeXatLxBI9gBaebbnrfifHhDYfgasaacH8akY=wiFfYdH8Gipec8Eeeu0xXdbba9frFj0=OqFfea0dXdd9vqai=hGuQ8kuc9pgc9s8qqaq=dirpe0xb9q8qiLsFr0=vr0=vr0dc8meaabaqaciaacaGaaeqabaqabeGadaaakeaafaqaaeGabaaabaWaaSaaaeaacqWGKbazcqWGybawaeaacqWGKbazcqWG0baDaaGaeyypa0JaemiwaGLaeiikaGIaeGymaeJaeyOeI0IaeiikaGIaemiEaGNaey4kaSIaemywaKLaeiykaKIaeiykaKIaeyOeI0Iaemyrau0aaSbaaSqaaGGaciab=j7aIbqabaGccqGGOaakcqWG0baDcqGGPaqkdaWcaaqaaiabdIfayjabdMfazbqaaiabdIfayjabgUcaRiabdMfazbaacqGGSaalaeaadaWcaaqaaiabdsgaKjabdMfazbqaaiabdsgaKjabdsha0baacqGH9aqpcqWFZoWzcqWGzbqwcqGGOaakcqaIXaqmcqGHsislcqGGOaakcqWGybawcqGHRaWkcqWGzbqwcqGGPaqkcqGGPaqkcqGHRaWkcqWGfbqrdaWgaaWcbaGae8NSdigabeaakiabcIcaOiabdsha0jabcMcaPmaalaaabaGaemiwaGLaemywaKfabaGaemiwaGLaey4kaSIaemywaKfaaiabgkHiTiab=r7aKjabdMfazjabcYcaSaaacaWLjaGaaCzcamaabmGabaGaeGynaudacaGLOaGaayzkaaaaaa@71F0@

where we suppressed the dependence of phase variables on *t*, and the initial conditions are *X*(0) = *X*_0_, *Y*(0) = *Y*_0_. In order to solve this system, we only need to know the explicit expression for *E*_*β *_(*t*). If we use the usual notation

Mβ(λ)=∫Beβλp(0,β)dβ
 MathType@MTEF@5@5@+=feaafiart1ev1aaatCvAUfKttLearuWrP9MDH5MBPbIqV92AaeXatLxBI9gBaebbnrfifHhDYfgasaacH8akY=wiFfYdH8Gipec8Eeeu0xXdbba9frFj0=OqFfea0dXdd9vqai=hGuQ8kuc9pgc9s8qqaq=dirpe0xb9q8qiLsFr0=vr0=vr0dc8meaabaqaciaacaGaaeqabaqabeGadaaakeaacqWGnbqtdaWgaaWcbaacciGae8NSdigabeaakiabcIcaOiab=T7aSjabcMcaPiabg2da9maapebabaGaemyzau2aaWbaaSqabeaacqWFYoGycqWF7oaBaaGccqWGWbaCcqGGOaakcqaIWaamcqGGSaalcqWFYoGycqGGPaqkcqWGKbazcqWFYoGyaSqaaiabbkeacbqab0Gaey4kIipaaaa@4570@

for the moment generation function (mgf) of the initial pdf, it can be shown ([61,62], see also MA) that the current mean value of *β *can be calculated using the mgf of the initial distribution of *β *; the exact expression is

Eβ(t)=1Mβ(q(t))dMβ(λ)dλ|λ=q(t),
 MathType@MTEF@5@5@+=feaafiart1ev1aaatCvAUfKttLearuWrP9MDH5MBPbIqV92AaeXatLxBI9gBaebbnrfifHhDYfgasaacH8akY=wiFfYdH8Gipec8Eeeu0xXdbba9frFj0=OqFfea0dXdd9vqai=hGuQ8kuc9pgc9s8qqaq=dirpe0xb9q8qiLsFr0=vr0=vr0dc8meaabaqaciaacaGaaeqabaqabeGadaaakeaadaabciqaaiabdweafnaaBaaaleaaiiGacqWFYoGyaeqaaOGaeiikaGIaemiDaqNaeiykaKIaeyypa0ZaaSaaaeaacqaIXaqmaeaacqWGnbqtdaWgaaWcbaGae8NSdigabeaakiabcIcaOiabdghaXjabcIcaOiabdsha0jabcMcaPiabcMcaPaaadaWcaaqaaiabdsgaKjabd2eannaaBaaaleaacqWFYoGyaeqaaOGaeiikaGIae83UdWMaeiykaKcabaGaemizaqMae83UdWgaaaGaayjcSdWaaSbaaSqaaiab=T7aSjabg2da9iabdghaXjabcIcaOiabdsha0jabcMcaPaqabaGccqGGSaalaaa@52A3@

where *q*(*t*) is an auxiliary variable that satisfies the equation

dqdt=YX+Y,q(0)=0,     (6)
 MathType@MTEF@5@5@+=feaafiart1ev1aaatCvAUfKttLearuWrP9MDH5MBPbIqV92AaeXatLxBI9gBaebbnrfifHhDYfgasaacH8akY=wiFfYdH8Gipec8Eeeu0xXdbba9frFj0=OqFfea0dXdd9vqai=hGuQ8kuc9pgc9s8qqaq=dirpe0xb9q8qiLsFr0=vr0=vr0dc8meaabaqaciaacaGaaeqabaqabeGadaaakeaafaqabeqacaaabaWaaSaaaeaacqWGKbazcqWGXbqCaeaacqWGKbazcqWG0baDaaGaeyypa0ZaaSaaaeaacqWGzbqwaeaacqWGybawcqGHRaWkcqWGzbqwaaGaeiilaWcabaGaemyCaeNaeiikaGIaeGimaaJaeiykaKIaeyypa0JaeGimaaJaeiilaWcaaiaaxMaacaWLjaWaaeWaceaacqaI2aGnaiaawIcacaGLPaaaaaa@4376@

and *M*_*β *_(λ) is given as far as we specify the initial pdf *p*(0, *β*).

In other words, if we want to keep track of the total population sizes in the distributed model (3)–(4), then this model implies the system of ODEs (5)–(6) that can be analyzed in the usual way; moreover, it is very easy to solve this system numerically (there are methods to obtain the solution of (3)–(4), e.g., [59], but these methods are usually computationally intensive and not so effective as numerical methods for ODEs). It is worth noting that the current population density *x*(*t*, *β*) also can be computed (see Theorem 1 in MA).

Note that system (5) differs from (2) in that the fixed value of parameter *β *is replaced by its current mean value *E*_*β *_(*t*) in the distributed model. This mean value, obviously, depends on the system dynamics and the population sizes (see (6) and above), and its calculation at any time moment gives us solution of problem (5).

It can be shown that, for model (3)–(4), the mean of the parameter distribution decreases monotonically with time (*dE*_*β *_(*t*)/*dt *≤ 0 for any *t*, see Corollary 2 in MA) from the initial value *E*_*β *_(0) to the final value η, which shows the direction of selection: the cells with lower parameter values are selected for. More importantly, however, the simultaneous knowledge of the time dependence of *E*_*β *_(*t*) and the parametric portrait of the homogeneous model (2) (Fig. 1) allows one to identify transient behavior of the solutions of (5) (and, accordingly, of model (3)), i.e., the behavior of solutions prior to the time moment when *E*_*β *_(*t*) becomes constant. This behavior can be of particular interest because this is one of the features that distinguishes model (3) from (2) and allows us to explore in detail the temporal dynamics of inhomogeneous biological systems.

Due to the non-negativity of *β*, we should consider only distributions with support on half-axis *β *≥ 0. To illustrate the possible dynamical behavior of the cell populations, we need the initial distribution of *β*, which is unknown in practice. In the following, we assume that the initial pdf of *β *is the gamma distribution on [η, ∞) with positive parameters *k*, *s *and η ≥ 0. The choice of the gamma distribution can be justified by the fact that it can well approximate almost any unimodal distribution concentrated on the positive half-line. Other possible initial distributions include uniform, log-normal, Pareto, and many others; any of them can be incorporated in the model as far as there exist mgfs for the given *q*(*t*) (we note, however, that, in some particular cases, mgf does not exist for some values of *q*(*t*), see MA).

For the case of gamma distributed *β *at the initial time moment, it can be proved (see MA, Example 1) that *β *is gamma-distributed at any time moment with *E*_*β *_(*t*) = η + *k */(*s-q*(*t*)). Typically, when *Y*(*t*) > *const *> 0, then *q*(*t*) → -∞ for *t *→ ∞ (see (6)) and hence *E*_*β *_(*t*) → η. Thus, the knowledge of the support of the initial distribution and the direction of selection allows us, in a number of cases, to predict the final state of the system.

In brief, we use the following approach to perform and present numerical simulations. First, we need to specify the initial conditions and the initial distribution of *β*, i.e., assign values of η, *k*, *s*. Here, for the sake of transparency, we have chosen to set the values of the mean *E*_*β *_= ∫Bβp(0,β)dβ
 MathType@MTEF@5@5@+=feaafiart1ev1aaatCvAUfKttLearuWrP9MDH5MBPbIqV92AaeXatLxBI9gBaebbnrfifHhDYfgasaacH8akY=wiFfYdH8Gipec8Eeeu0xXdbba9frFj0=OqFfea0dXdd9vqai=hGuQ8kuc9pgc9s8qqaq=dirpe0xb9q8qiLsFr0=vr0=vr0dc8meaabaqaciaacaGaaeqabaqabeGadaaakeaadaWdraqaaGGaciab=j7aIjabdchaWjabcIcaOiabicdaWiabcYcaSiab=j7aIjabcMcaPiabdsgaKjab=j7aIbWcbaGaeeOqaieabeqdcqGHRiI8aaaa@3ADB@ and variance σβ2
 MathType@MTEF@5@5@+=feaafiart1ev1aaatCvAUfKttLearuWrP9MDH5MBPbIqV92AaeXatLxBI9gBaebbnrfifHhDYfgasaacH8akY=wiFfYdH8Gipec8Eeeu0xXdbba9frFj0=OqFfea0dXdd9vqai=hGuQ8kuc9pgc9s8qqaq=dirpe0xb9q8qiLsFr0=vr0=vr0dc8meaabaqaciaacaGaaeqabaqabeGadaaakeaaiiGacqWFdpWCdaqhaaWcbaGae8NSdigabaGaeGOmaidaaaaa@3131@ = ∫Bβ2p(0,β)dβ
 MathType@MTEF@5@5@+=feaafiart1ev1aaatCvAUfKttLearuWrP9MDH5MBPbIqV92AaeXatLxBI9gBaebbnrfifHhDYfgasaacH8akY=wiFfYdH8Gipec8Eeeu0xXdbba9frFj0=OqFfea0dXdd9vqai=hGuQ8kuc9pgc9s8qqaq=dirpe0xb9q8qiLsFr0=vr0=vr0dc8meaabaqaciaacaGaaeqabaqabeGadaaakeaadaWdraqaaGGaciab=j7aInaaCaaaleqabaGaeGOmaidaaOGaemiCaaNaeiikaGIaeGimaaJaeiilaWIae8NSdiMaeiykaKIaemizaqMae8NSdigaleaacqqGcbGqaeqaniabgUIiYdaaaa@3C04@ - *E*_*β *_(0)^2 ^of the initial distribution, as well as the bounds of the distribution if they are different from zero or infinity (for the gamma distribution, the bound that has to be specified is its left boundary, η). In the analyzed case (when we work with the distribution that has two free parameters, *k *and *s*), this is sufficient to unambiguously determine the values of the parameters (obviously, however, this is not the case in the general situation).

Loosely speaking, during the simulation, the parametric point with coordinates (*γ*, *δ*, *E*_*β *_(*t*)) travels in the parameter space of model (2); the movement occurs along the line connecting points (*γ*, *δ*, *E*_*β *_(0)) and (*γ*, *δ*, η) in the parameter portrait of (2) (see Fig. 1 for the cut of the parameter space for fixed *γ*). The speed of movement is determined, together with the initial conditions *X*_0 _and *Y*_0_, by the parameters of the initial distribution, or, equivalently, by the initial mean and variance of the gamma distribution with the given left boundary.

**Figure 2:**
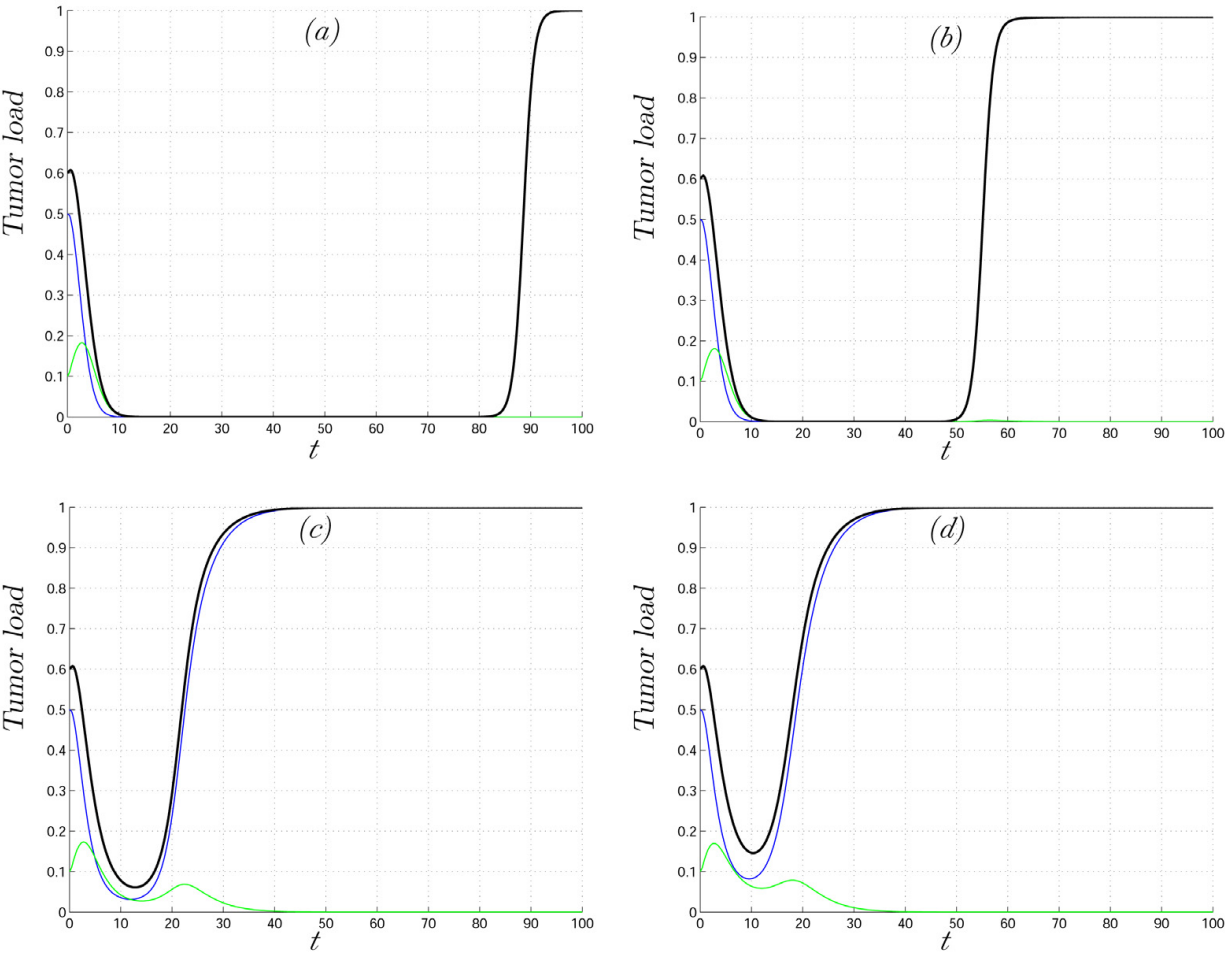
Solutions of system (5)–(6) with gamma distributed parameter *β *on [1.5, ∞). Uninfected cells, *X*(*t*), infected cells, *Y*(*t*), and the total tumor load, *X*(*t*) + *Y*(*t*), are shown in blue, green and black, respectively. The initial conditions *X*(0) = 0.5, *Y*(0) = 0.1, parameter values *γ *= 1, *δ *= 2. The initial mean of distribution *E*_*β *_(0) = 2.5, the initial variances 0.06 (a), 0.1(b), 0.3(c), 0.4(d).

Results of several numerical simulations of system (5)–(6) are shown in Fig. 2. The parameter values were chosen such that we start in domain *VIII *(Fig. 1) (eradication of the tumor), cross domain *VII *(bistable situation), and end up in domain *I *(no effect of virus therapy). The solutions shown in Fig. 2 reflect the fact that the degree of heterogeneity plays an important role in the model dynamics. The parameter values and initial conditions are the same for all four simulations; the difference comes from different initial variances of *β *; the greater the initial variance the faster we reach the unfavorable domain *I*. Conversely, the initial variance of the distribution can be small enough such that the time during which the size of the tumor remains negligible (*X *+ *Y *is close to zero) is comparable with the life-time of a patient; this emphasizes that we need to know not only the final state of *E*_*β *_(*t*) but also its transient behavior.

Fig. 2 shows the phenomenon of tumor recurrence after a relatively long period of small tumor load, a phenomenon that is not seen in the original homogeneous model (2). Clearly, reappearance of the tumor is a probable outcome of tumor-specific virus treatment if there are cancer cells that are inaccessible to the virus (or are accessible at an extremely slow rate compared to the characteristic time of virus propagation): after the virus kills off the susceptible tumor cells and is cleared (given that it cannot infect healthy tissues), these resistant cancer cells can develop into a new tumor. It should be emphasized that, in the simulations in Fig. 2, the final outcome is tumor recurrence, i.e., the tumor dynamics is strongly affected by cell heterogeneity although all cells have a positive probability to be infected (η > 0). This example shows that heterogeneous model (3), together with dynamical regimes inherited from model (2), possesses new regimes, and, thus, is more general.

**Figure 3:**
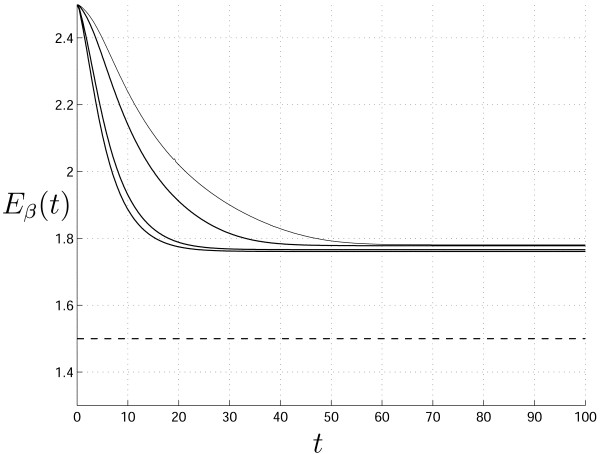
*E*_*β *_(*t*) versus time for the cases presented in Fig. 2. The curves from top to bottom correspond to panels (a)–(d) in Fig. 2.

The change of the mean parameter value for the cases presented in Fig. 2 is shown in Fig. 3. It can be seen that higher rate of change corresponds to greater values of the initial variance; in addition, in none of the analyzed cases *E*_*β *_(*t*) reaches the final value η, because the speed of movement is determined by two simultaneously acting factors: the characteristics of the mgf and the equation for the auxiliary variable *q*(*t*) for which *dq*(*t*)/*dt *≈ 0 starting from some time *t*.

**Figure 4:**
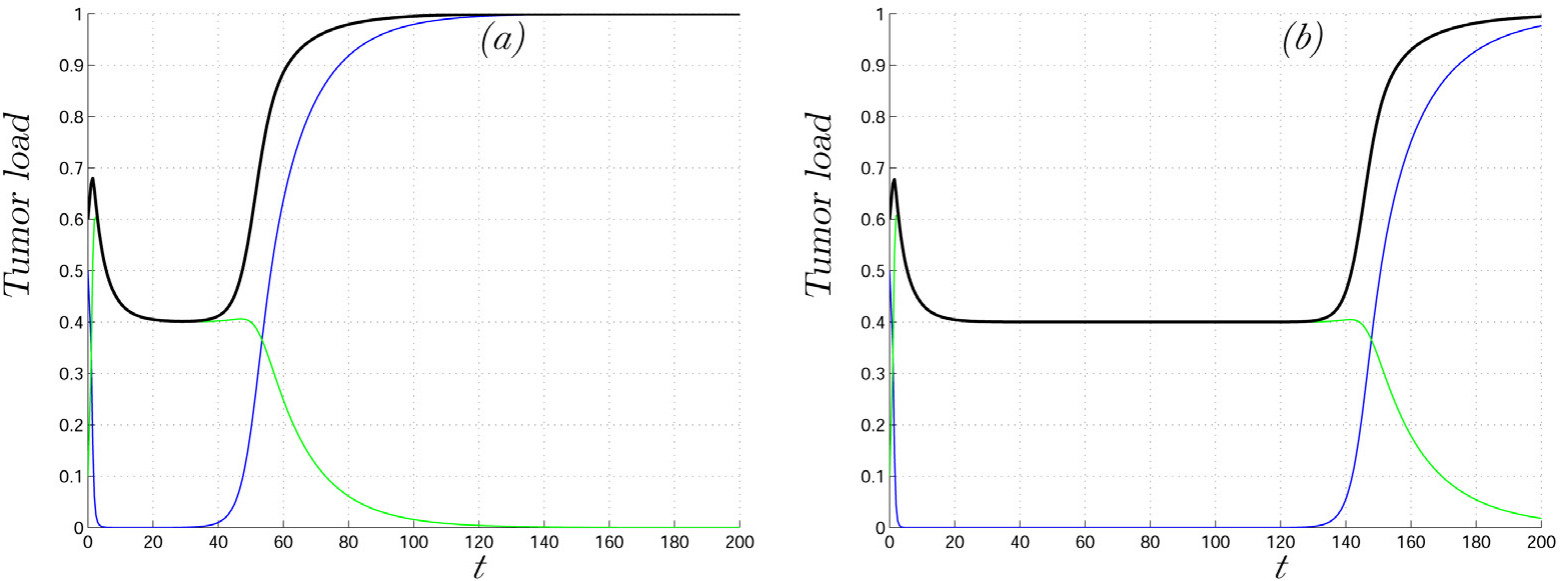
Solutions of system (5)–(6) with gamma distributed parameter *β *on [0.1, ∞). Uninfected cells, *X*(*t*), infected cells, *Y*(*t*), and the total tumor load, *X*(*t*) + *Y*(*t*), are shown in blue, green, and black, respectively. The initial conditions are *X*(0) = 0.5, *Y*(0) = 0.1, parameter values *γ *= 0.5, *δ *= 0.3. The initial mean of distribution *E*_*β *_(0) = 3.5, the initial variances 1.5 (a) and 0.5(b).

Other possible cases can be similarly analyzed; for example, if we choose parameters such that the starting point belongs to domain *IV *(Fig. 1a) and the final point belongs to domain *II*, it is not difficult to predict that, first, there will be a short period of time when the tumor load remains constant, then the tumor starts growing linearly (when *E*_*β *_(*t*) belongs to domain *V*), and in domain *II*, the tumor grows under the logistic law (Fig. 4). Thus, accommodation of heterogeneity results in model dynamics that reflects the phenomenon of temporary dormancy of the tumor (Fig. 4).

#### Distributed susceptibility and distributed cytotoxicity

Together with differential susceptibility considered in the previous section, parameter *δ *(the rate of killing of infected cells) also may be assumed to take different values in the infected cell population. The population of infected cells can be heterogeneous on its own such that some cells are more likely to die faster than others when infected by the virus. From different perspective, we can attribute this heterogeneity to the virus, i.e., the virus population is assumed to consist of strains that differ in their ability to kill cells. In previous work [51,52], it has been shown that, in some parameter domains, there is an optimal level of cytotoxicity that is necessary to maximally reduce the tumor size if other parameter values are fixed. In addition, it is sometimes desirable to kill as many cells as possible during a short period of time; such situation would favor using different viruses for therapeutic purposes. Moreover, the latter assumption on virus heterogeneity can help one to interpret the model equations which, assuming that *δ *takes values from set Δ, and *y*(*t*, *δ*) is the density of infected cells having parameter value *δ*, take the form:

∂x(t,β)∂t=x(t,β)[1−(X(t)+Y(t))]−βx(t,β)Y(t)X(t)+y(t),∂y(t,δ)∂t=γy(t,δ)[1−(X(t)+Y(t))]+Eβ(t)X(t)y(t,δ)X(t)+Y(t)−δy(t,δ),     (7)
 MathType@MTEF@5@5@+=feaafiart1ev1aaatCvAUfKttLearuWrP9MDH5MBPbIqV92AaeXatLxBI9gBaebbnrfifHhDYfgasaacH8akY=wiFfYdH8Gipec8Eeeu0xXdbba9frFj0=OqFfea0dXdd9vqai=hGuQ8kuc9pgc9s8qqaq=dirpe0xb9q8qiLsFr0=vr0=vr0dc8meaabaqaciaacaGaaeqabaqabeGadaaakeaafaqaaeGabaaabaWaaSaaaeaacqGHciITcqWG4baEcqGGOaakcqWG0baDcqGGSaaliiGacqWFYoGycqGGPaqkaeaacqGHciITcqWG0baDaaGaeyypa0JaemiEaGNaeiikaGIaemiDaqNaeiilaWIae8NSdiMaeiykaKIaei4waSLaeGymaeJaeyOeI0IaeiikaGIaemiwaGLaeiikaGIaemiDaqNaeiykaKIaey4kaSIaemywaKLaeiikaGIaemiDaqNaeiykaKIaeiykaKIaeiyxa0LaeyOeI0YaaSaaaeaacqWFYoGycqWG4baEcqGGOaakcqWG0baDcqGGSaalcqWFYoGycqGGPaqkcqWGzbqwcqGGOaakcqWG0baDcqGGPaqkaeaacqWGybawcqGGOaakcqWG0baDcqGGPaqkcqGHRaWkcqWG5bqEcqGGOaakcqWG0baDcqGGPaqkaaGaeiilaWcabaWaaSaaaeaacqGHciITcqWG5bqEcqGGOaakcqWG0baDcqGGSaalcqWF0oazcqGGPaqkaeaacqGHciITcqWG0baDaaGaeyypa0Jae83SdCMaemyEaKNaeiikaGIaemiDaqNaeiilaWIae8hTdqMaeiykaKIaei4waSLaeGymaeJaeyOeI0IaeiikaGIaemiwaGLaeiikaGIaemiDaqNaeiykaKIaey4kaSIaemywaKLaeiikaGIaemiDaqNaeiykaKIaeiykaKIaeiyxa0Laey4kaSYaaSaaaeaacqWGfbqrdaWgaaWcbaGae8NSdigabeaakiabcIcaOiabdsha0jabcMcaPiabdIfayjabcIcaOiabdsha0jabcMcaPiabdMha5jabcIcaOiabdsha0jabcYcaSiab=r7aKjabcMcaPaqaaiabdIfayjabcIcaOiabdsha0jabcMcaPiabgUcaRiabdMfazjabcIcaOiabdsha0jabcMcaPaaacqGHsislcqWF0oazcqWG5bqEcqGGOaakcqWG0baDcqGGSaalcqWF0oazcqGGPaqkcqGGSaalaaGaaCzcaiaaxMaadaqadiqaaiabiEda3aGaayjkaiaawMcaaaaa@B7FF@

where *E*_*β *_(*t*) is defined as above, and the initial conditions are

*x*(0, *β*) = *x*_0_(*β*) = *X*_0 _*p*_1 _(0, *β*), *y*(0, *δ*) = *y*_0_(*δ*) = *Y*_0 _*p*_2 _(0, *δ*).      (8)

Note that in (7) both cell populations are closed under reproduction; that is, e.g., the uninfected cell that has parameter value β^* ^can produce daughter cells only with the same parameter value (the same holds for the infected cell population), moreover, if an uninfected cell was infected by the virus from an infected cell with a parameter value δ^*^, it falls into the same class. If we assume that *δ *is an attribute of the cell population, this assumption is difficult to justify, whereas if *δ *is an attribute of the virus, the assumption follows from obvious considerations.

System (7)–(8) implies the following system of ODEs (see MA, Corollary 1):

dXdt=X(1−(X+Y))−Eβ(t)XYX+Y,dYdt=γY(1−(X+Y))+Eβ(t)XYX+Y−Eδ(t)Y,X(0)=X0,Y(0)=Y0     (9)
 MathType@MTEF@5@5@+=feaafiart1ev1aaatCvAUfKttLearuWrP9MDH5MBPbIqV92AaeXatLxBI9gBaebbnrfifHhDYfgasaacH8akY=wiFfYdH8Gipec8Eeeu0xXdbba9frFj0=OqFfea0dXdd9vqai=hGuQ8kuc9pgc9s8qqaq=dirpe0xb9q8qiLsFr0=vr0=vr0dc8meaabaqaciaacaGaaeqabaqabeGadaaakeaafaqaaeWabaaabaWaaSaaaeaacqWGKbazcqWGybawaeaacqWGKbazcqWG0baDaaGaeyypa0JaemiwaGLaeiikaGIaeGymaeJaeyOeI0IaeiikaGIaemiwaGLaey4kaSIaemywaKLaeiykaKIaeiykaKIaeyOeI0Iaemyrau0aaSbaaSqaaGGaciab=j7aIbqabaGccqGGOaakcqWG0baDcqGGPaqkdaWcaaqaaiabdIfayjabdMfazbqaaiabdIfayjabgUcaRiabdMfazbaacqGGSaalaeaadaWcaaqaaiabdsgaKjabdMfazbqaaiabdsgaKjabdsha0baacqGH9aqpcqWFZoWzcqWGzbqwcqGGOaakcqaIXaqmcqGHsislcqGGOaakcqWGybawcqGHRaWkcqWGzbqwcqGGPaqkcqGGPaqkcqGHRaWkcqWGfbqrdaWgaaWcbaGae8NSdigabeaakiabcIcaOiabdsha0jabcMcaPmaalaaabaGaemiwaGLaemywaKfabaGaemiwaGLaey4kaSIaemywaKfaaiabgkHiTiabdweafnaaBaaaleaacqWF0oazaeqaaOGaeiikaGIaemiDaqNaeiykaKIaemywaKLaeiilaWcabaqbaeqabeGaaaqaaiabdIfayjabcIcaOiabicdaWiabcMcaPiabg2da9iabdIfaynaaBaaaleaacqaIWaamaeqaaOGaeiilaWcabaGaemywaKLaeiikaGIaeGimaaJaeiykaKIaeyypa0JaemywaK1aaSbaaSqaaiabicdaWaqabaaaaaaakiaaxMaacaWLjaWaaeWaceaacqaI5aqoaiaawIcacaGLPaaaaaa@858F@

where the mean parameter values are

Eβ(t)=1Mβ(q1(t))dMβ(λ)dλ|λ=q1(t),Eδ(t)=1Mδ(q2(t))dMδ(λ)dλ|λ=q2(t),
 MathType@MTEF@5@5@+=feaafiart1ev1aaatCvAUfKttLearuWrP9MDH5MBPbIqV92AaeXatLxBI9gBaebbnrfifHhDYfgasaacH8akY=wiFfYdH8Gipec8Eeeu0xXdbba9frFj0=OqFfea0dXdd9vqai=hGuQ8kuc9pgc9s8qqaq=dirpe0xb9q8qiLsFr0=vr0=vr0dc8meaabaqaciaacaGaaeqabaqabeGadaaakeaafaqabeqacaaabaWaaqGaceaacqWGfbqrdaWgaaWcbaacciGae8NSdigabeaakiabcIcaOiabdsha0jabcMcaPiabg2da9maalaaabaGaeGymaedabaGaemyta00aaSbaaSqaaiab=j7aIbqabaGccqGGOaakcqWGXbqCdaWgaaWcbaGaeGymaedabeaakiabcIcaOiabdsha0jabcMcaPiabcMcaPaaadaWcaaqaaiabdsgaKjabd2eannaaBaaaleaacqWFYoGyaeqaaOGaeiikaGIae83UdWMaeiykaKcabaGaemizaqMae83UdWgaaaGaayjcSdWaaSbaaSqaaiab=T7aSjabg2da9iabdghaXnaaBaaameaacqaIXaqmaeqaaSGaeiikaGIaemiDaqNaeiykaKcabeaakiabcYcaSaqaamaaeiGabaGaemyrau0aaSbaaSqaaiab=r7aKbqabaGccqGGOaakcqWG0baDcqGGPaqkcqGH9aqpdaWcaaqaaiabigdaXaqaaiabd2eannaaBaaaleaacqWF0oazaeqaaOGaeiikaGIaemyCae3aaSbaaSqaaiabikdaYaqabaGccqGGOaakcqWG0baDcqGGPaqkcqGGPaqkaaWaaSaaaeaacqWGKbazcqWGnbqtdaWgaaWcbaGae8hTdqgabeaakiabcIcaOiab=T7aSjabcMcaPaqaaiabdsgaKjab=T7aSbaaaiaawIa7amaaBaaaleaacqWF7oaBcqGH9aqpcqWGXbqCdaWgaaadbaGaeGOmaidabeaaliabcIcaOiabdsha0jabcMcaPaqabaaaaOGaeiilaWcaaa@7D47@

for the given mgf of *p*_1 _(0, *β*) and *p*_2_(0, *δ*), and the auxiliary variables can be found from

dq1dt=−YX+Y,dq2dt=−1,q1(0)=0,q2(0)=0.     (10)
 MathType@MTEF@5@5@+=feaafiart1ev1aaatCvAUfKttLearuWrP9MDH5MBPbIqV92AaeXatLxBI9gBaebbnrfifHhDYfgasaacH8akY=wiFfYdH8Gipec8Eeeu0xXdbba9frFj0=OqFfea0dXdd9vqai=hGuQ8kuc9pgc9s8qqaq=dirpe0xb9q8qiLsFr0=vr0=vr0dc8meaabaqaciaacaGaaeqabaqabeGadaaakeaafaqabeqadaaabaWaaSaaaeaacqWGKbazcqWGXbqCdaWgaaWcbaGaeGymaedabeaaaOqaaiabdsgaKjabdsha0baacqGH9aqpcqGHsisldaWcaaqaaiabdMfazbqaaiabdIfayjabgUcaRiabdMfazbaacqGGSaalaeaadaWcaaqaaiabdsgaKjabdghaXnaaBaaaleaacqaIYaGmaeqaaaGcbaGaemizaqMaemiDaqhaaiabg2da9iabgkHiTiabigdaXiabcYcaSaqaaiabdghaXnaaBaaaleaacqaIXaqmaeqaaOGaeiikaGIaeGimaaJaeiykaKIaeyypa0JaeGimaaJaeiilaWIaemyCae3aaSbaaSqaaiabikdaYaqabaGccqGGOaakcqaIWaamcqGGPaqkcqGH9aqpcqaIWaamaaGaeiOla4IaaCzcaiaaxMaadaqadiqaaiabigdaXiabicdaWaGaayjkaiaawMcaaaaa@5A19@

To analyze system (9)–(10), one has to specify initial conditions and initial distributions for parameters *β *and *δ*. As before, to obtain some insight into the possible dynamical behavior of (9), we can consider the parameter portrait (Fig. 1) and time-dependent changes of the mean parameter values. In the case under consideration *E*_*β *_(*t*), as before, moves from top to bottom in Fig. 1, whereas *E*_*δ *_(*t*) goes from right to left. Note that the speed of movement in the *δ *-direction is determined only by the properties of the mgf, because *q*_2 _(*t*) = -*t *from (10) and *q*_2_(*t*) does not depend on the population sizes.

**Figure 5:**
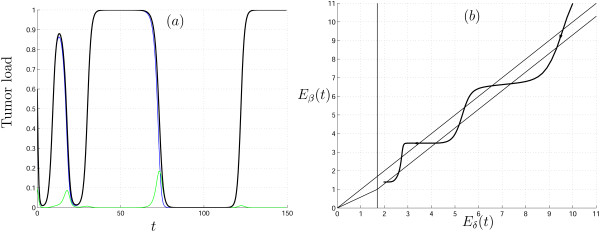
(a) Solutions of system (9)–(10) with gamma distributed parameters *β *on [1, ∞) and *δ *on [1, ∞). Uninfected cells, *X*(*t*), infected cells, *Y*(*t*), and the total tumor load, *X*(*t*) + *Y*(*t*), are shown in blue, green and black, respectively. The initial means of the distributions are *E*_*β *_(0) = 11 and *E*_*δ *_(0) = 10, initial variances are σβ2
 MathType@MTEF@5@5@+=feaafiart1ev1aaatCvAUfKttLearuWrP9MDH5MBPbIqV92AaeXatLxBI9gBaebbnrfifHhDYfgasaacH8akY=wiFfYdH8Gipec8Eeeu0xXdbba9frFj0=OqFfea0dXdd9vqai=hGuQ8kuc9pgc9s8qqaq=dirpe0xb9q8qiLsFr0=vr0=vr0dc8meaabaqaciaacaGaaeqabaqabeGadaaakeaaiiGacqWFdpWCdaqhaaWcbaGae8NSdigabaGaeGOmaidaaaaa@3131@ (0) = 8 and σδ2
 MathType@MTEF@5@5@+=feaafiart1ev1aaatCvAUfKttLearuWrP9MDH5MBPbIqV92AaeXatLxBI9gBaebbnrfifHhDYfgasaacH8akY=wiFfYdH8Gipec8Eeeu0xXdbba9frFj0=OqFfea0dXdd9vqai=hGuQ8kuc9pgc9s8qqaq=dirpe0xb9q8qiLsFr0=vr0=vr0dc8meaabaqaciaacaGaaeqabaqabeGadaaakeaaiiGacqWFdpWCdaqhaaWcbaGae8hTdqgabaGaeGOmaidaaaaa@3135@ (0) = 0.5. The initial conditions are *X*(0) = 0.5, *Y*(0) = 0.1, and *γ *= 1. (b) The parametric curve (*E*_*β *_(*t*), *E*_*δ *_(*t*)) in the parameter space (compare with Fig. 1b)

**Figure 6:**
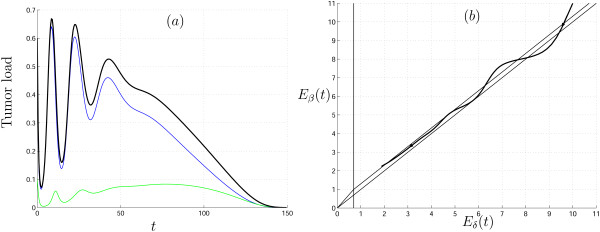
(a) Solutions of system (9)–(10) with gamma-distributed parameters *β *on [2, ∞) and *δ *on [0.9, ∞). Uninfected cells, *X*(*t*), infected cells, *Y*(*t*), and the total tumor load, *X*(*t*) + *Y*(*t*), are shown in blue, green and black, respectively. The initial means of distributions are *E*_*β *_(0) = 11 and *E*_*δ *_(0) = 10, initial variances are σβ2
 MathType@MTEF@5@5@+=feaafiart1ev1aaatCvAUfKttLearuWrP9MDH5MBPbIqV92AaeXatLxBI9gBaebbnrfifHhDYfgasaacH8akY=wiFfYdH8Gipec8Eeeu0xXdbba9frFj0=OqFfea0dXdd9vqai=hGuQ8kuc9pgc9s8qqaq=dirpe0xb9q8qiLsFr0=vr0=vr0dc8meaabaqaciaacaGaaeqabaqabeGadaaakeaaiiGacqWFdpWCdaqhaaWcbaGae8NSdigabaGaeGOmaidaaaaa@3131@ (0) = 8 and σδ2
 MathType@MTEF@5@5@+=feaafiart1ev1aaatCvAUfKttLearuWrP9MDH5MBPbIqV92AaeXatLxBI9gBaebbnrfifHhDYfgasaacH8akY=wiFfYdH8Gipec8Eeeu0xXdbba9frFj0=OqFfea0dXdd9vqai=hGuQ8kuc9pgc9s8qqaq=dirpe0xb9q8qiLsFr0=vr0=vr0dc8meaabaqaciaacaGaaeqabaqabeGadaaakeaaiiGacqWFdpWCdaqhaaWcbaGae8hTdqgabaGaeGOmaidaaaaa@3135@ (0) = 0.5. The initial conditions *X*(0) = 0.5, *Y*(0) = 0.1 and *γ *= 0.7. (b) The parametric curve (*E*_*β *_(*t*), *E*_*δ *_(*t*)) in the parameter space

Generally, the transient behavior of cell populations (prior to reaching the final state – if it is ever reached) can be quite complex. This can be seen in Fig. 4 where the solutions of (9)–(10) are shown (Fig. 5a), and the path of the mean parameter values depicted (Fig. 5b). Due to the structure of the parametric portrait of (2), the mean parameter values can pass through domains of qualitatively different behavior many times, thus resulting in complex and unpredictable picture. Moreover, even if the initial and final parameter points belong to the same domain, during transient process, the mean parameter values can visit other domains (Fig. 6).

In Fig. 6, the initial and final parameter values belong to the most favorable domain *VIII *(Fig. 1), in which complete eradication of the tumor cells occurs. On its way to extinction, however, the tumor load behaves in an irregular, complex way. This example shows the possibility that complex and erratic observational data can be explained not only by random effects and noise but also by the innate heterogeneity of the cell and virus populations.

In general, both distributed susceptibility of uninfected cells and distributed cytotoxicity of the virus do not favor the tumor treatment and decrease chances for effective cure by increasing tumor robustness; one of the possible ways to overcome this problem (within the framework of the considered models) is suggested in the next section.

#### Distributed susceptibility and distributed virulence

The inherent heterogeneity of tumor cells implies that cells differ in their susceptibility to virus infection, and this situation was modeled in two previous sections. In general, it can be assumed that the virus population is also heterogeneous in the sense that it contains viruses with different ability to infect tumor cells, which we denote virulence. Inasmuch as model (1) does not explicitly model the virus dynamics, we can incorporate the effect of virus heterogeneity into the infected cell population. Assuming the susceptibility of uninfected cells *β*_1 _and the virus virulence *β*_2 _to be independent, we can consider the transmission coefficient *β *as being proportional to the product of *β*_1 _and *β*_2 _(moreover, the proportionality constant can be incorporated in one of the parameters, thus we have *β *= *β*_1_*β*_2_).

As before, denote B_1 _the set of possible values of *β*_1 _and *x*(*t*, *β*_1_) the density of uninfected cells having the given value of *β*_1 _at the moment *t*; similarly, denote B_2 _the set of possible values of *β*_2 _and *y*(*t*, *β*_2_) the density of infected cells with the given value of *β*_2 _at the time moment *t*. The number of cells with susceptibility *β*_1 _newly infected by the virus produced from previously infected cells with virulence *β*_2 _is given by *β*_1_*β*_2_*x*(*t*, *β*_1_) *y *(*t*, *β*_2_)/(*X*(*t*) + *Y*(*t*)). The total number of cells that leave the uninfected population and have susceptibility *β*_1 _is *β*_1_*x*(*t*, *β*_1_) ∫B2β2y(t,β2)dβ2
 MathType@MTEF@5@5@+=feaafiart1ev1aaatCvAUfKttLearuWrP9MDH5MBPbIqV92AaeXatLxBI9gBaebbnrfifHhDYfgasaacH8akY=wiFfYdH8Gipec8Eeeu0xXdbba9frFj0=OqFfea0dXdd9vqai=hGuQ8kuc9pgc9s8qqaq=dirpe0xb9q8qiLsFr0=vr0=vr0dc8meaabaqaciaacaGaaeqabaqabeGadaaakeaadaWdraqaaGGaciab=j7aInaaBaaaleaacqaIYaGmaeqaaOGaemyEaKNaeiikaGIaemiDaqNaeiilaWIae8NSdi2aaSbaaSqaaiabikdaYaqabaGccqGGPaqkcqWGKbazcqWFYoGydaWgaaWcbaGaeGOmaidabeaaaeaacqqGcbGqdaWgaaadbaGaeGOmaidabeaaaSqab0Gaey4kIipaaaa@3FFD@/(*X*(*t*) + *Y*(*t*)); and the total change in the infected cell population with parameter value *β*_2 _due to the infection process is given by *β*_2 _*y*(*t*, *β*_2_) ∫B1β1x(t,β1)dβ1
 MathType@MTEF@5@5@+=feaafiart1ev1aaatCvAUfKttLearuWrP9MDH5MBPbIqV92AaeXatLxBI9gBaebbnrfifHhDYfgasaacH8akY=wiFfYdH8Gipec8Eeeu0xXdbba9frFj0=OqFfea0dXdd9vqai=hGuQ8kuc9pgc9s8qqaq=dirpe0xb9q8qiLsFr0=vr0=vr0dc8meaabaqaciaacaGaaeqabaqabeGadaaakeaadaWdraqaaGGaciab=j7aInaaBaaaleaacqaIXaqmaeqaaOGaemiEaGNaeiikaGIaemiDaqNaeiilaWIae8NSdi2aaSbaaSqaaiabigdaXaqabaGccqGGPaqkcqWGKbazcqWFYoGydaWgaaWcbaGaeGymaedabeaaaeaacqqGcbGqdaWgaaadbaGaeGymaedabeaaaSqab0Gaey4kIipaaaa@3FF3@/(*X*(*t*) + *Y*(*t*)). Combining the above assumptions, we obtain the model

∂x(t,β1)∂t=x(t,β1)[1−(X(t)+Y(t))]−β1x(t,β1)Eβ2(t)Y(t)X(t)+Y(t),∂y(t,β2)∂t=γy(t,β2)[1−(X(t)+Y(t))]+β2y(t,β2)Eβ1(t)X(t)X(t)+Y(t)−δy(t,β2),     (11)
 MathType@MTEF@5@5@+=feaafiart1ev1aaatCvAUfKttLearuWrP9MDH5MBPbIqV92AaeXatLxBI9gBaebbnrfifHhDYfgasaacH8akY=wiFfYdH8Gipec8Eeeu0xXdbba9frFj0=OqFfea0dXdd9vqai=hGuQ8kuc9pgc9s8qqaq=dirpe0xb9q8qiLsFr0=vr0=vr0dc8meaabaqaciaacaGaaeqabaqabeGadaaakeaafaqabeGabaaabaWaaSaaaeaacqGHciITcqWG4baEcqGGOaakcqWG0baDcqGGSaaliiGacqWFYoGydaWgaaWcbaGaeGymaedabeaakiabcMcaPaqaaiabgkGi2kabdsha0baacqGH9aqpcqWG4baEcqGGOaakcqWG0baDcqGGSaalcqWFYoGydaWgaaWcbaGaeGymaedabeaakiabcMcaPiabcUfaBjabigdaXiabgkHiTiabcIcaOiabdIfayjabcIcaOiabdsha0jabcMcaPiabgUcaRiabdMfazjabcIcaOiabdsha0jabcMcaPiabcMcaPiabc2faDjabgkHiTmaalaaabaGae8NSdi2aaSbaaSqaaiabigdaXaqabaGccqWG4baEcqGGOaakcqWG0baDcqGGSaalcqWFYoGydaWgaaWcbaGaeGymaedabeaakiabcMcaPiabdweafnaaBaaaleaacqWFYoGydaWgaaadbaGaeGOmaidabeaaaSqabaGccqGGOaakcqWG0baDcqGGPaqkcqWGzbqwcqGGOaakcqWG0baDcqGGPaqkaeaacqWGybawcqGGOaakcqWG0baDcqGGPaqkcqGHRaWkcqWGzbqwcqGGOaakcqWG0baDcqGGPaqkaaGaeiilaWcabaWaaSaaaeaacqGHciITcqWG5bqEcqGGOaakcqWG0baDcqGGSaalcqWFYoGydaWgaaWcbaGaeGOmaidabeaakiabcMcaPaqaaiabgkGi2kabdsha0baacqGH9aqpcqWFZoWzcqWG5bqEcqGGOaakcqWG0baDcqGGSaalcqWFYoGydaWgaaWcbaGaeGOmaidabeaakiabcMcaPiabcUfaBjabigdaXiabgkHiTiabcIcaOiabdIfayjabcIcaOiabdsha0jabcMcaPiabgUcaRiabdMfazjabcIcaOiabdsha0jabcMcaPiabcMcaPiabc2faDjabgUcaRmaalaaabaGae8NSdi2aaSbaaSqaaiabikdaYaqabaGccqWG5bqEcqGGOaakcqWG0baDcqGGSaalcqWFYoGydaWgaaWcbaGaeGOmaidabeaakiabcMcaPiabdweafnaaBaaaleaacqWFYoGydaWgaaadbaGaeGymaedabeaaaSqabaGccqGGOaakcqWG0baDcqGGPaqkcqWGybawcqGGOaakcqWG0baDcqGGPaqkaeaacqWGybawcqGGOaakcqWG0baDcqGGPaqkcqGHRaWkcqWGzbqwcqGGOaakcqWG0baDcqGGPaqkaaGaeyOeI0Iae8hTdqMaemyEaKNaeiikaGIaemiDaqNaeiilaWIae8NSdi2aaSbaaSqaaiabikdaYaqabaGccqGGPaqkcqGGSaalaaGaaCzcaiaaxMaadaqadiqaaiabigdaXiabigdaXaGaayjkaiaawMcaaaaa@CCEA@

where the notations Eβ1
 MathType@MTEF@5@5@+=feaafiart1ev1aaatCvAUfKttLearuWrP9MDH5MBPbIqV92AaeXatLxBI9gBaebbnrfifHhDYfgasaacH8akY=wiFfYdH8Gipec8Eeeu0xXdbba9frFj0=OqFfea0dXdd9vqai=hGuQ8kuc9pgc9s8qqaq=dirpe0xb9q8qiLsFr0=vr0=vr0dc8meaabaqaciaacaGaaeqabaqabeGadaaakeaacqWGfbqrdaWgaaWcbaacciGae8NSdi2aaSbaaWqaaiabigdaXaqabaaaleqaaaaa@30BB@ (*t*) = (∫B1β1x(t,β1)dβ1
 MathType@MTEF@5@5@+=feaafiart1ev1aaatCvAUfKttLearuWrP9MDH5MBPbIqV92AaeXatLxBI9gBaebbnrfifHhDYfgasaacH8akY=wiFfYdH8Gipec8Eeeu0xXdbba9frFj0=OqFfea0dXdd9vqai=hGuQ8kuc9pgc9s8qqaq=dirpe0xb9q8qiLsFr0=vr0=vr0dc8meaabaqaciaacaGaaeqabaqabeGadaaakeaadaWdraqaaGGaciab=j7aInaaBaaaleaacqaIXaqmaeqaaOGaemiEaGNaeiikaGIaemiDaqNaeiilaWIae8NSdi2aaSbaaSqaaiabigdaXaqabaGccqGGPaqkcqWGKbazcqWFYoGydaWgaaWcbaGaeGymaedabeaaaeaacqqGcbGqdaWgaaadbaGaeGymaedabeaaaSqab0Gaey4kIipaaaa@3FF3@)/*X*(*t*), Eβ2
 MathType@MTEF@5@5@+=feaafiart1ev1aaatCvAUfKttLearuWrP9MDH5MBPbIqV92AaeXatLxBI9gBaebbnrfifHhDYfgasaacH8akY=wiFfYdH8Gipec8Eeeu0xXdbba9frFj0=OqFfea0dXdd9vqai=hGuQ8kuc9pgc9s8qqaq=dirpe0xb9q8qiLsFr0=vr0=vr0dc8meaabaqaciaacaGaaeqabaqabeGadaaakeaacqWGfbqrdaWgaaWcbaacciGae8NSdi2aaSbaaWqaaiabikdaYaqabaaaleqaaaaa@30BD@ (*t*) = (∫B2β2y(t,β2)dβ2
 MathType@MTEF@5@5@+=feaafiart1ev1aaatCvAUfKttLearuWrP9MDH5MBPbIqV92AaeXatLxBI9gBaebbnrfifHhDYfgasaacH8akY=wiFfYdH8Gipec8Eeeu0xXdbba9frFj0=OqFfea0dXdd9vqai=hGuQ8kuc9pgc9s8qqaq=dirpe0xb9q8qiLsFr0=vr0=vr0dc8meaabaqaciaacaGaaeqabaqabeGadaaakeaadaWdraqaaGGaciab=j7aInaaBaaaleaacqaIYaGmaeqaaOGaemyEaKNaeiikaGIaemiDaqNaeiilaWIae8NSdi2aaSbaaSqaaiabikdaYaqabaGccqGGPaqkcqWGKbazcqWFYoGydaWgaaWcbaGaeGOmaidabeaaaeaacqqGcbGqdaWgaaadbaGaeGOmaidabeaaaSqab0Gaey4kIipaaaa@3FFD@)/*Y*(*t*) were used for the current mean values of the corresponding parameters, and the initial conditions are

*x*(0, *β*_1_) = *x*_0_(*β*_1_) = *X*_0 _*p*_1_(0, *β*_1_), *y*(0, *β*_2_) = *y*_0_(*β*_2_) = *Y*_0 _*p*_2_(0, *β*_2_),       (12)

The model (11)–(12) implies the system of ODEs (Corollary 1, MA)

dXdt=X(1−(X+Y))−E(t)XYX+Y,dYdt=γY(1−(X+Y))+E(t)XYX+Y−δY,X(0)=X0,Y(0)=Y0     (13)
 MathType@MTEF@5@5@+=feaafiart1ev1aaatCvAUfKttLearuWrP9MDH5MBPbIqV92AaeXatLxBI9gBaebbnrfifHhDYfgasaacH8akY=wiFfYdH8Gipec8Eeeu0xXdbba9frFj0=OqFfea0dXdd9vqai=hGuQ8kuc9pgc9s8qqaq=dirpe0xb9q8qiLsFr0=vr0=vr0dc8meaabaqaciaacaGaaeqabaqabeGadaaakeaafaqaaeWabaaabaWaaSaaaeaacqWGKbazcqWGybawaeaacqWGKbazcqWG0baDaaGaeyypa0JaemiwaGLaeiikaGIaeGymaeJaeyOeI0IaeiikaGIaemiwaGLaey4kaSIaemywaKLaeiykaKIaeiykaKIaeyOeI0IaemyrauKaeiikaGIaemiDaqNaeiykaKYaaSaaaeaacqWGybawcqWGzbqwaeaacqWGybawcqGHRaWkcqWGzbqwaaGaeiilaWcabaWaaSaaaeaacqWGKbazcqWGzbqwaeaacqWGKbazcqWG0baDaaGaeyypa0dcciGae83SdCMaemywaKLaeiikaGIaeGymaeJaeyOeI0IaeiikaGIaemiwaGLaey4kaSIaemywaKLaeiykaKIaeiykaKIaey4kaSIaemyrauKaeiikaGIaemiDaqNaeiykaKYaaSaaaeaacqWGybawcqWGzbqwaeaacqWGybawcqGHRaWkcqWGzbqwaaGaeyOeI0Iae8hTdqMaemywaKLaeiilaWcabaqbaeqabeGaaaqaaiabdIfayjabcIcaOiabicdaWiabcMcaPiabg2da9iabdIfaynaaBaaaleaacqaIWaamaeqaaOGaeiilaWcabaGaemywaKLaeiikaGIaeGimaaJaeiykaKIaeyypa0JaemywaK1aaSbaaSqaaiabicdaWaqabaaaaaaakiaaxMaacaWLjaWaaeWaceaacqaIXaqmcqaIZaWmaiaawIcacaGLPaaaaaa@7E63@

where the mean parameter value is *E*(*t*) = Eβ1
 MathType@MTEF@5@5@+=feaafiart1ev1aaatCvAUfKttLearuWrP9MDH5MBPbIqV92AaeXatLxBI9gBaebbnrfifHhDYfgasaacH8akY=wiFfYdH8Gipec8Eeeu0xXdbba9frFj0=OqFfea0dXdd9vqai=hGuQ8kuc9pgc9s8qqaq=dirpe0xb9q8qiLsFr0=vr0=vr0dc8meaabaqaciaacaGaaeqabaqabeGadaaakeaacqWGfbqrdaWgaaWcbaacciGae8NSdi2aaSbaaWqaaiabigdaXaqabaaaleqaaaaa@30BB@ (*t*) Eβ2
 MathType@MTEF@5@5@+=feaafiart1ev1aaatCvAUfKttLearuWrP9MDH5MBPbIqV92AaeXatLxBI9gBaebbnrfifHhDYfgasaacH8akY=wiFfYdH8Gipec8Eeeu0xXdbba9frFj0=OqFfea0dXdd9vqai=hGuQ8kuc9pgc9s8qqaq=dirpe0xb9q8qiLsFr0=vr0=vr0dc8meaabaqaciaacaGaaeqabaqabeGadaaakeaacqWGfbqrdaWgaaWcbaacciGae8NSdi2aaSbaaWqaaiabikdaYaqabaaaleqaaaaa@30BD@ (*t*), and

Eβ1(t)=1Mβ1(q1(t))dMβ1(λ)dλ|λ=q1(t),Eβ2(t)=1Mβ2(q2(t))dMβ2(λ)dλ|λ=q2(t)
 MathType@MTEF@5@5@+=feaafiart1ev1aaatCvAUfKttLearuWrP9MDH5MBPbIqV92AaeXatLxBI9gBaebbnrfifHhDYfgasaacH8akY=wiFfYdH8Gipec8Eeeu0xXdbba9frFj0=OqFfea0dXdd9vqai=hGuQ8kuc9pgc9s8qqaq=dirpe0xb9q8qiLsFr0=vr0=vr0dc8meaabaqaciaacaGaaeqabaqabeGadaaakeaafaqabeqacaaabaWaaqGaceaacqWGfbqrdaWgaaWcbaacciGae8NSdi2aaSbaaWqaaiabbgdaXaqabaaaleqaaOGaeiikaGIaemiDaqNaeiykaKIaeyypa0ZaaSaaaeaacqaIXaqmaeaacqWGnbqtdaWgaaWcbaGae8NSdi2aaSbaaWqaaiabbgdaXaqabaaaleqaaOGaeiikaGIaemyCae3aaSbaaSqaaiabigdaXaqabaGccqGGOaakcqWG0baDcqGGPaqkcqGGPaqkaaWaaSaaaeaacqWGKbazcqWGnbqtdaWgaaWcbaGae8NSdi2aaSbaaWqaaiabbgdaXaqabaaaleqaaOGaeiikaGIae83UdWMaeiykaKcabaGaemizaqMae83UdWgaaaGaayjcSdWaaSbaaSqaaiab=T7aSjabg2da9iabdghaXnaaBaaameaacqaIXaqmaeqaaSGaeiikaGIaemiDaqNaeiykaKcabeaakiabcYcaSaqaamaaeiGabaGaemyrau0aaSbaaSqaaiab=j7aInaaBaaameaacqqGYaGmaeqaaaWcbeaakiabcIcaOiabdsha0jabcMcaPiabg2da9maalaaabaGaeGymaedabaGaemyta00aaSbaaSqaaiab=j7aInaaBaaameaacqqGYaGmaeqaaaWcbeaakiabcIcaOiabdghaXnaaBaaaleaacqaIYaGmaeqaaOGaeiikaGIaemiDaqNaeiykaKIaeiykaKcaamaalaaabaGaemizaqMaemyta00aaSbaaSqaaiab=j7aInaaBaaameaacqqGYaGmaeqaaaWcbeaakiabcIcaOiab=T7aSjabcMcaPaqaaiabdsgaKjab=T7aSbaaaiaawIa7amaaBaaaleaacqWF7oaBcqGH9aqpcqWGXbqCdaWgaaadbaGaeGOmaidabeaaliabcIcaOiabdsha0jabcMcaPaqabaaaaaaa@831D@

are expressed with the mgfs of *p*_1 _(0, *β*_1_), *p*_2 _(0, *β*_2_). The auxiliary variables can be found from the equations

dq1dt=−Eβ2(t)YX+Y,dq2dt=Eβ1(t)XX+Y,q1(0)=0, q2(0)=0     (14)
 MathType@MTEF@5@5@+=feaafiart1ev1aaatCvAUfKttLearuWrP9MDH5MBPbIqV92AaeXatLxBI9gBaebbnrfifHhDYfgasaacH8akY=wiFfYdH8Gipec8Eeeu0xXdbba9frFj0=OqFfea0dXdd9vqai=hGuQ8kuc9pgc9s8qqaq=dirpe0xb9q8qiLsFr0=vr0=vr0dc8meaabaqaciaacaGaaeqabaqabeGadaaakeaafaqaaeGabaaabaqbaeaabeGaaaqaamaalaaabaGaemizaqMaemyCae3aaSbaaSqaaiabigdaXaqabaaakeaacqWGKbazcqWG0baDaaGaeyypa0JaeyOeI0Iaemyrau0aaSbaaSqaaGGaciab=j7aInaaBaaameaacqaIYaGmaeqaaaWcbeaakiabcIcaOiabdsha0jabcMcaPmaalaaabaGaemywaKfabaGaemiwaGLaey4kaSIaemywaKfaaiabcYcaSaqaamaalaaabaGaemizaqMaemyCae3aaSbaaSqaaiabikdaYaqabaaakeaacqWGKbazcqWG0baDaaGaeyypa0Jaemyrau0aaSbaaSqaaiab=j7aInaaBaaameaacqaIXaqmaeqaaaWcbeaakiabcIcaOiabdsha0jabcMcaPmaalaaabaGaemiwaGfabaGaemiwaGLaey4kaSIaemywaKfaaiabcYcaSaaaaeaacqWGXbqCdaWgaaWcbaGaeGymaedabeaakiabcIcaOiabicdaWiabcMcaPiabg2da9iabicdaWiabcYcaSiabbccaGiabdghaXnaaBaaaleaacqaIYaGmaeqaaOGaeiikaGIaeGimaaJaeiykaKIaeyypa0JaeGimaadaaiaaxMaacaWLjaWaaeWaceaacqaIXaqmcqaI0aanaiaawIcacaGLPaaaaaa@6B3D@

As before, we have to specify the initial pdfs for *β*_1 _and *β*_2 _Now we have *dq*_2 _(*t*)/*dt *≥ 0, and it can be shown that *d*Eβ2
 MathType@MTEF@5@5@+=feaafiart1ev1aaatCvAUfKttLearuWrP9MDH5MBPbIqV92AaeXatLxBI9gBaebbnrfifHhDYfgasaacH8akY=wiFfYdH8Gipec8Eeeu0xXdbba9frFj0=OqFfea0dXdd9vqai=hGuQ8kuc9pgc9s8qqaq=dirpe0xb9q8qiLsFr0=vr0=vr0dc8meaabaqaciaacaGaaeqabaqabeGadaaakeaacqWGfbqrdaWgaaWcbaacciGae8NSdi2aaSbaaWqaaiabikdaYaqabaaaleqaaaaa@30BD@ (*t*)/*dt *≥ 0 (Corollary 2, MA), that is, formally, the movement in the *β*_2_-direction goes from smaller to greater mean values. In order not to deal with an infinitely large coefficient, it is reasonable to assume that the initial distribution of *β*_2 _is determined on a closed interval [*c*_1_, *c*_2_]. In this case, we can choose as the initial distribution the beta distribution on [*c*_1_, *c*_2_].

**Figure 7:**
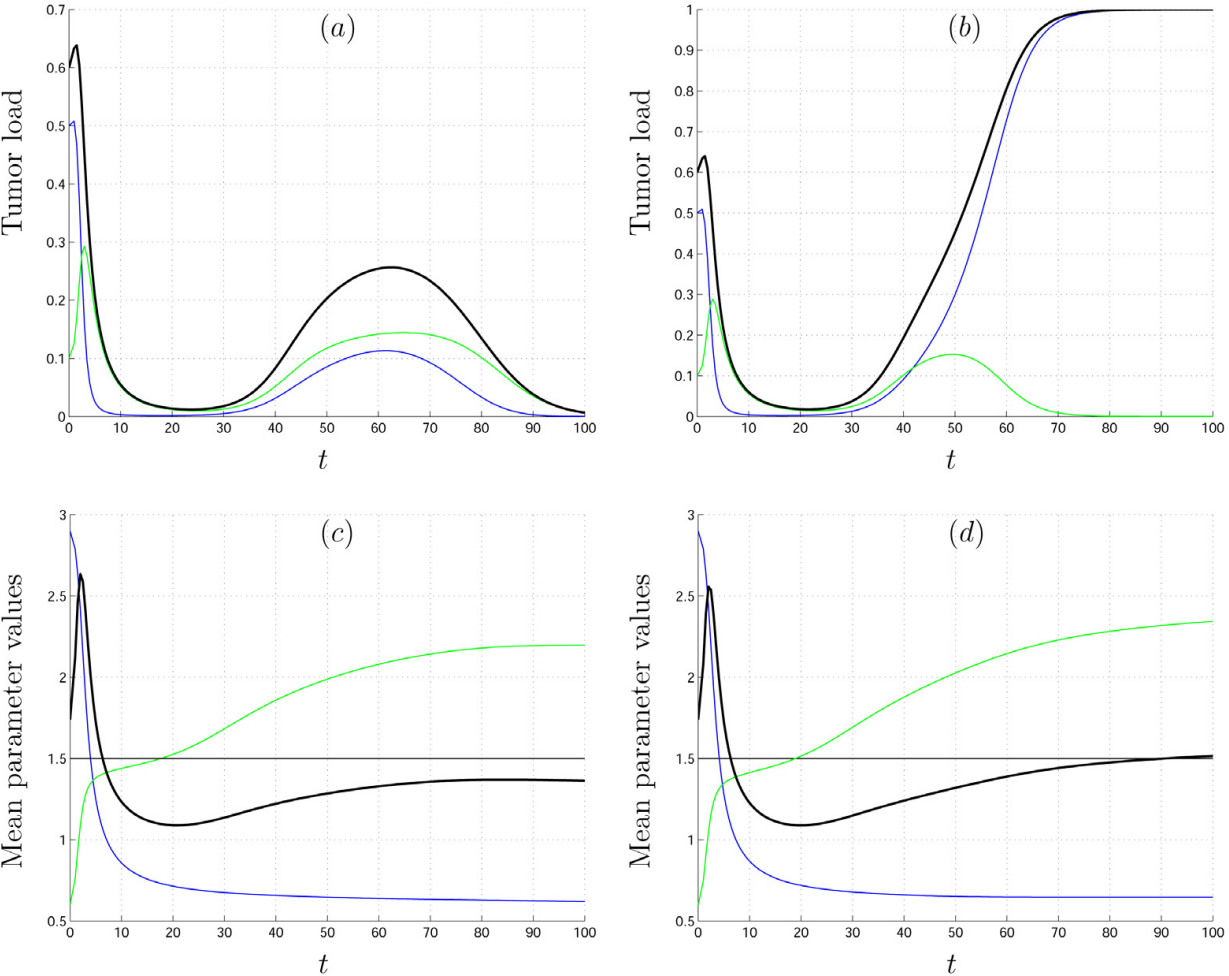
Solutions of system (13)–(14) with both uninfected cell specific and infected cell specific distributions of transmission coefficient *β*. *β*_1 _is gamma-distributed on [0.6, ∞) and *β*_2 _is beta-distributed on [0,2.5]. Uninfected cells, *X*(*t*), infected cells, *Y*(*t*), and the total tumor load, *X*(*t*) + *Y*(*t*), are shown in blue, green and black, respectively. The initial means of distributions are Eβ1
 MathType@MTEF@5@5@+=feaafiart1ev1aaatCvAUfKttLearuWrP9MDH5MBPbIqV92AaeXatLxBI9gBaebbnrfifHhDYfgasaacH8akY=wiFfYdH8Gipec8Eeeu0xXdbba9frFj0=OqFfea0dXdd9vqai=hGuQ8kuc9pgc9s8qqaq=dirpe0xb9q8qiLsFr0=vr0=vr0dc8meaabaqaciaacaGaaeqabaqabeGadaaakeaacqWGfbqrdaWgaaWcbaacciGae8NSdi2aaSbaaWqaaiabigdaXaqabaaaleqaaaaa@30BB@ (0) = 2.9 and Eβ2
 MathType@MTEF@5@5@+=feaafiart1ev1aaatCvAUfKttLearuWrP9MDH5MBPbIqV92AaeXatLxBI9gBaebbnrfifHhDYfgasaacH8akY=wiFfYdH8Gipec8Eeeu0xXdbba9frFj0=OqFfea0dXdd9vqai=hGuQ8kuc9pgc9s8qqaq=dirpe0xb9q8qiLsFr0=vr0=vr0dc8meaabaqaciaacaGaaeqabaqabeGadaaakeaacqWGfbqrdaWgaaWcbaacciGae8NSdi2aaSbaaWqaaiabikdaYaqabaaaleqaaaaa@30BD@ (0) = 0.6, initial variances are σβ12
 MathType@MTEF@5@5@+=feaafiart1ev1aaatCvAUfKttLearuWrP9MDH5MBPbIqV92AaeXatLxBI9gBaebbnrfifHhDYfgasaacH8akY=wiFfYdH8Gipec8Eeeu0xXdbba9frFj0=OqFfea0dXdd9vqai=hGuQ8kuc9pgc9s8qqaq=dirpe0xb9q8qiLsFr0=vr0=vr0dc8meaabaqaciaacaGaaeqabaqabeGadaaakeaaiiGacqWFdpWCdaqhaaWcbaGae8NSdi2aaSbaaWqaaiabigdaXaqabaaaleaacqaIYaGmaaaaaa@3259@ (0) = 1.9 and σβ22
 MathType@MTEF@5@5@+=feaafiart1ev1aaatCvAUfKttLearuWrP9MDH5MBPbIqV92AaeXatLxBI9gBaebbnrfifHhDYfgasaacH8akY=wiFfYdH8Gipec8Eeeu0xXdbba9frFj0=OqFfea0dXdd9vqai=hGuQ8kuc9pgc9s8qqaq=dirpe0xb9q8qiLsFr0=vr0=vr0dc8meaabaqaciaacaGaaeqabaqabeGadaaakeaaiiGacqWFdpWCdaqhaaWcbaGae8NSdi2aaSbaaWqaaiabikdaYaqabaaaleaacqaIYaGmaaaaaa@325B@ (0) = 0.12 (a) σβ22
 MathType@MTEF@5@5@+=feaafiart1ev1aaatCvAUfKttLearuWrP9MDH5MBPbIqV92AaeXatLxBI9gBaebbnrfifHhDYfgasaacH8akY=wiFfYdH8Gipec8Eeeu0xXdbba9frFj0=OqFfea0dXdd9vqai=hGuQ8kuc9pgc9s8qqaq=dirpe0xb9q8qiLsFr0=vr0=vr0dc8meaabaqaciaacaGaaeqabaqabeGadaaakeaaiiGacqWFdpWCdaqhaaWcbaGae8NSdi2aaSbaaWqaaiabikdaYaqabaaaleaacqaIYaGmaaaaaa@325B@ (0) = 0.11 (b). In panels (c) and (d), the mean parameter values Eβ1
 MathType@MTEF@5@5@+=feaafiart1ev1aaatCvAUfKttLearuWrP9MDH5MBPbIqV92AaeXatLxBI9gBaebbnrfifHhDYfgasaacH8akY=wiFfYdH8Gipec8Eeeu0xXdbba9frFj0=OqFfea0dXdd9vqai=hGuQ8kuc9pgc9s8qqaq=dirpe0xb9q8qiLsFr0=vr0=vr0dc8meaabaqaciaacaGaaeqabaqabeGadaaakeaacqWGfbqrdaWgaaWcbaacciGae8NSdi2aaSbaaWqaaiabigdaXaqabaaaleqaaaaa@30BB@ (*t*), Eβ2
 MathType@MTEF@5@5@+=feaafiart1ev1aaatCvAUfKttLearuWrP9MDH5MBPbIqV92AaeXatLxBI9gBaebbnrfifHhDYfgasaacH8akY=wiFfYdH8Gipec8Eeeu0xXdbba9frFj0=OqFfea0dXdd9vqai=hGuQ8kuc9pgc9s8qqaq=dirpe0xb9q8qiLsFr0=vr0=vr0dc8meaabaqaciaacaGaaeqabaqabeGadaaakeaacqWGfbqrdaWgaaWcbaacciGae8NSdi2aaSbaaWqaaiabikdaYaqabaaaleqaaaaa@30BD@ (*t*) and *E*(*t*) = Eβ1
 MathType@MTEF@5@5@+=feaafiart1ev1aaatCvAUfKttLearuWrP9MDH5MBPbIqV92AaeXatLxBI9gBaebbnrfifHhDYfgasaacH8akY=wiFfYdH8Gipec8Eeeu0xXdbba9frFj0=OqFfea0dXdd9vqai=hGuQ8kuc9pgc9s8qqaq=dirpe0xb9q8qiLsFr0=vr0=vr0dc8meaabaqaciaacaGaaeqabaqabeGadaaakeaacqWGfbqrdaWgaaWcbaacciGae8NSdi2aaSbaaWqaaiabigdaXaqabaaaleqaaaaa@30BB@ (*t*) Eβ2
 MathType@MTEF@5@5@+=feaafiart1ev1aaatCvAUfKttLearuWrP9MDH5MBPbIqV92AaeXatLxBI9gBaebbnrfifHhDYfgasaacH8akY=wiFfYdH8Gipec8Eeeu0xXdbba9frFj0=OqFfea0dXdd9vqai=hGuQ8kuc9pgc9s8qqaq=dirpe0xb9q8qiLsFr0=vr0=vr0dc8meaabaqaciaacaGaaeqabaqabeGadaaakeaacqWGfbqrdaWgaaWcbaacciGae8NSdi2aaSbaaWqaaiabikdaYaqabaaaleqaaaaa@30BD@ (*t*) are shown for cases (a) and (b), respectively

To get insight into the transient behavior of the model solutions, we have to consider the product of the mean parameter values. Again, we move along the line in the *β*-direction (Fig. 1), starting from Eβ1
 MathType@MTEF@5@5@+=feaafiart1ev1aaatCvAUfKttLearuWrP9MDH5MBPbIqV92AaeXatLxBI9gBaebbnrfifHhDYfgasaacH8akY=wiFfYdH8Gipec8Eeeu0xXdbba9frFj0=OqFfea0dXdd9vqai=hGuQ8kuc9pgc9s8qqaq=dirpe0xb9q8qiLsFr0=vr0=vr0dc8meaabaqaciaacaGaaeqabaqabeGadaaakeaacqWGfbqrdaWgaaWcbaacciGae8NSdi2aaSbaaWqaaiabigdaXaqabaaaleqaaaaa@30BB@ (0) Eβ2
 MathType@MTEF@5@5@+=feaafiart1ev1aaatCvAUfKttLearuWrP9MDH5MBPbIqV92AaeXatLxBI9gBaebbnrfifHhDYfgasaacH8akY=wiFfYdH8Gipec8Eeeu0xXdbba9frFj0=OqFfea0dXdd9vqai=hGuQ8kuc9pgc9s8qqaq=dirpe0xb9q8qiLsFr0=vr0=vr0dc8meaabaqaciaacaGaaeqabaqabeGadaaakeaacqWGfbqrdaWgaaWcbaacciGae8NSdi2aaSbaaWqaaiabikdaYaqabaaaleqaaaaa@30BD@ (0), with the asymptotic state η*c*_2_. The important difference now is that the function *E*(*t*) does not have to be monotonic (Fig. 7).

In Fig. 7, the results of numerical simulation of system (13)–(14) are shown together with functions Eβ1
 MathType@MTEF@5@5@+=feaafiart1ev1aaatCvAUfKttLearuWrP9MDH5MBPbIqV92AaeXatLxBI9gBaebbnrfifHhDYfgasaacH8akY=wiFfYdH8Gipec8Eeeu0xXdbba9frFj0=OqFfea0dXdd9vqai=hGuQ8kuc9pgc9s8qqaq=dirpe0xb9q8qiLsFr0=vr0=vr0dc8meaabaqaciaacaGaaeqabaqabeGadaaakeaacqWGfbqrdaWgaaWcbaacciGae8NSdi2aaSbaaWqaaiabigdaXaqabaaaleqaaaaa@30BB@ (*t*), Eβ2
 MathType@MTEF@5@5@+=feaafiart1ev1aaatCvAUfKttLearuWrP9MDH5MBPbIqV92AaeXatLxBI9gBaebbnrfifHhDYfgasaacH8akY=wiFfYdH8Gipec8Eeeu0xXdbba9frFj0=OqFfea0dXdd9vqai=hGuQ8kuc9pgc9s8qqaq=dirpe0xb9q8qiLsFr0=vr0=vr0dc8meaabaqaciaacaGaaeqabaqabeGadaaakeaacqWGfbqrdaWgaaWcbaacciGae8NSdi2aaSbaaWqaaiabikdaYaqabaaaleqaaaaa@30BD@ (*t*) and *E*(*t*) = Eβ1
 MathType@MTEF@5@5@+=feaafiart1ev1aaatCvAUfKttLearuWrP9MDH5MBPbIqV92AaeXatLxBI9gBaebbnrfifHhDYfgasaacH8akY=wiFfYdH8Gipec8Eeeu0xXdbba9frFj0=OqFfea0dXdd9vqai=hGuQ8kuc9pgc9s8qqaq=dirpe0xb9q8qiLsFr0=vr0=vr0dc8meaabaqaciaacaGaaeqabaqabeGadaaakeaacqWGfbqrdaWgaaWcbaacciGae8NSdi2aaSbaaWqaaiabigdaXaqabaaaleqaaaaa@30BB@ (*t*) Eβ2
 MathType@MTEF@5@5@+=feaafiart1ev1aaatCvAUfKttLearuWrP9MDH5MBPbIqV92AaeXatLxBI9gBaebbnrfifHhDYfgasaacH8akY=wiFfYdH8Gipec8Eeeu0xXdbba9frFj0=OqFfea0dXdd9vqai=hGuQ8kuc9pgc9s8qqaq=dirpe0xb9q8qiLsFr0=vr0=vr0dc8meaabaqaciaacaGaaeqabaqabeGadaaakeaacqWGfbqrdaWgaaWcbaacciGae8NSdi2aaSbaaWqaaiabikdaYaqabaaaleqaaaaa@30BD@ (*t*). We start in domain *VIII *(elliptic sector in Fig. 1), and the asymptotic state is in domain *VII *where two opposite outcomes are possible, namely, complete tumor eradication or logistic tumor growth. The initial conditions and parameter values are the same for both cases; the two cases differ only in the initial variance of the *β*_2 _distribution. It can be seen from Fig. 7 that even a small difference in the variance of *β*_2 _may yield dramatically different results: in the first case, the tumor is cured, whereas, in the second case, virus therapy fails. This example emphasizes that, to predict the outcome of oncolytic virus therapy, it is necessary to know not only the initial sizes of the cell populations but also the degree of heterogeneity of all the parameters under consideration.

## Discussion and conclusions

We presented a general framework within which to construct and analyze mathematical models of anticancer treatment, with a special emphasis on tumor heterogeneity. Conceptually, this approach is connected to the theoretical considerations of Kitano on cancer robustness [1,2]. At this initial step of analysis, it appears most important that the mathematical models reproduce important behaviors of tumors at the qualitative level. Using the previously developed model of tumor cell-oncolytic virus interaction [51], we show that qualitatively distinct behaviors that are absent in the homogeneous model appear as natural consequences of tumor and oncolytic virus heterogeneity. For example, our model accounts for cancer recurrence, and the time until reappearance of the tumor crucially depends on the heterogeneity of the transmission coefficient (Fig. 2). It should be emphasized that the effects of the tumor cell heterogeneity are not limited to trivial resistance of a subpopulation of cells to the virus, i.e., recurrence might ensue even when all cells have a non-zero probability to be infected. Another phenomenon missing in the homogeneous model is constant tumor size during a particular period of time (Fig. 4). Heterogeneity in parameters can lead to complex, irregular evolution of the tumor (Fig. 5 and 6). Thus, interpretation of the results of anticancer therapy should take into account the possibility that irregular, quasi-chaotic behavior can be caused not only by random fluctuations but also by the heterogeneity of the tumor and the virus (Fig. 6). All the simulations presented here reveal the effect of the level of heterogeneity on tumor dynamics. The most obvious case is shown in Fig. 7 where different initial variances of parameter distribution lead either to tumor eradication or to logistic tumor growth, i.e., failure of virus therapy.

Analysis of the models proves that tumor heterogeneity increases cancer robustness, in agreement with the theoretical considerations of Kitano [2]. The results presented here further show that, to counter this adverse effect of tumor heterogeneity, it should be possible to employ a heterogeneous population of an oncolytic virus (see model (11) and Figure 7). The interaction of the two non-homogeneous populations, the tumor and the oncolytic virus, may result in complete elimination of the tumor.

We applied a previously developed, general mathematical technique [61,62] to investigate non-homogeneous models of complex tumor cells-virus interaction. The advantages of this modeling approach are as follows. This approach can account for different types of parametric heterogeneity of the analyzed populations. To infer the consequences of heterogeneity, we use well-known mathematical tools, such as bifurcation analysis, which identifies points of qualitative change in the system dynamics. The theory of heterogeneous populations [61,62,65] allows one to reduce the models to systems of ODEs that, in many cases, can be explored analytically or, if this is not possible, can be solved numerically with high precision.

Cancer is an evolving system, and the main evolutionary forces are selection, mutation and random drift. It should be noticed that our approach explicitly examines only selection. The techniques is applied to deterministic systems, and it is our belief that such system are important for modeling purposes, although analysis of extinction phenomena, such as tumor eradication, may require stochastic factors to be considered. The principal obstacle to the validation of these models against empirical data and their use for prediction purposes is the necessity to know the initial distribution of the model parameters. It should be emphasized that knowledge of mean, variance, and any finite number of the moments of the distribution is not sufficient to describe the evolution of the system over indefinite time [62]. The entire distributions have to be known. However, there is a way to bypass this problem. First, knowledge of several first moments is sufficient to estimate tumor evolution over short time spans. Second, to model the evolutionary process, we need to know how the mean parameter values behave with time. Using the theory of heterogeneous populations, we can infer generic properties of these functions and identify them from empirical data [66].

To conclude, the approach developed here allows one to take stock of such a complex aspect of cancer as tumor heterogeneity and apply effective analytical techniques to the analysis of heterogeneous models of tumor evolution. Although, in this study, we analyzed the specific case of a tumor interaction with an oncolytic virus, it is our hope that these techniques will prove useful in other systems that include interaction between tumor cells and anticancer agents.

## Authors' contributions

GPK and ASN developed the mathematical models; EVK incepted the study and provided the biological interpretations; ASN wrote the initial draft of the paper, GPK wrote the Mathematical Appendix, and EVK produced the final version of the article; all authors edited and approved the manuscript.

## Reviewers' comments

### Reviewer's report 1

Leonid Hanin, Department of Mathematics, Idaho State University (nominated by Arcady Mushegian)

## Comments for the authors

The paper deals with mathematical modeling of a novel mode of cancer therapy whereby a tumor is treated with oncolytic viruses that have a selective affinity to cancer cells. The model is couched as a system of coupled differential equations for the sizes of populations of uninfected an infected cancer cells. The main thrust of the work is on introducing parametric heterogeneity and analytic/numerical study of its effects on the dynamics of tumor cell populations. The problems in questions are of significant theoretical and practical interest. The authors suggested a few compelling general ideas, demonstrated a good knowledge of both biomedical and mathematical aspects of the general field, and provided ample references.

However, the paper concerns me on several levels.

1. The model was derived not from a detailed theoretical or experimental study of the underlying biological phenomena but rather from "general considerations." Also, it was not fitted to any set of real data on the sizes of tumor cell populations interacting with oncolytic viruses *in vivo *or *in vitro*. Thus, the model lacks biological specificity, and its adequacy has not been established. In particular, is there any strong evidence of the assumed heterogeneity of tumor cell populations with regard to killing action of oncolytic viruses, and does this assumption improve the quality of the model and its predictive power?

**Author response**: *The model was developed to describe qualitative behaviors of the system "tumor cells – oncolytic virus", and we by no means claim that this model should be used for quantitative predictions. The current empirical data is insufficient to obtain robust estimates of the model parameters. These points are addressed in greater detail in our previous paper in this series (Ref*. [51]*)*.

*The hypothesis that tumor cell populations are heterogeneous with regard to the killing action of oncolytic viruses is a general one, and our model strongly suggests that the assumption of ****homogeneity****(that is, that all the tumor cells behave in the same way under oncolytic virus infection) is an oversimplification. Indeed, there is no strong evidence that tumors are heterogeneous in terms of their response to oncolytic virus infection but this seems highly likely given the well-demonstrated heterogeneity of tumors in other respects, in particular, chemotherapy. We investigate the implications of heterogeneity for the outcome of oncolytic virus treatment and hope that our model stimulates experimental studies in this direction*.

2. The way parametric heterogeneity was introduced has a fundamental flaw. Speaking about the distribution *x*_*β *_of the susceptibility parameter *β *(that is, the rate of virus transmission from infected to uninfected tumor cells) in the population of uninfected tumor cells of size *X*, the authors assumed that the distribution of *β *is a function of time t alone. In reality, this distribution depends on the population size *X*:*x*_*β *_= *x*_*β *_(·, *X*) or more generally, *x*_*β *_= *x*_*β *_(·, *X*, *t*). For example, even in the simplest case of a uniformly distributed *β*, its density is given by *X*/|*B*|1_*B *_and thus depends on *X*. Omission of the dependence of the distribution of *β *on *X *is tantamount to assuming that every first order ODE is of the form *y *= *f*(*t*), where t is independent variable and y is unknown function, on the grounds that for each solution the right-hand side of such an ODE is a function of *t*. As a result, the model of distributed susceptibility, which in reality is non-linear, is trivialized to a linear model which, naturally, boils down to the time course of the expected value of parameter *β*. Treatment of the heterogeneity of other parameters (cytotoxicity and virulence) suffers from the same defect.

**Author response**: *This is, in our opinion, the main objection of the reviewer to the subject and conclusions of our work, and the only one with which we completely disagree*.

*i) We never stated or assumed *"that the distribution of *β *is a function of time t alone"; *as a matter of fact, we do not make any explicit assumptions on the distribution dynamics except for the given initial distribution and the form of the model equations. Under the proposed mathematical formalism (Theorem 1 in MA [see *Additional file 1*]) and equations used to describe the system, we can unambiguously determine the evolution of the parameter distribution given the initial distribution. In particular, we never assumed that the parameter (β) is gamma-distributed at any time moment with a time-dependent mean and variance; on the contrary, we suppose only that β is gamma-distributed at the initial moment and prove that then it must be gamma-distributed at any time moment due to the system dynamics, and compute its mean and variance depending on time (Theorem 1 and Example 1, MA [see *Additional file 1*])*.

*ii) We emphasize that x*_*β *_*is not a probability density function, that is, its integral equals the total population size and not 1. Theorem 1 and its corollary show how one can calculate the density x*_*β *_*, the total population size X*(*t*)*, the probability density function x*_*β*_/*X(t) and its moment generation function. To calculate all these quantities we use nonlinear systems ((A.4)–(A.6) in MA [see *Additional file 1*]) which evidently depend on the current population sizes in a complex way. The same is true for all other parameters and versions of the model*.

3. The model formulation contains arbitrary assumptions that are not justified and at times not even explicitly stated:

(a) The authors assume logistic growth of the sizes *X *and *Y *of infected and uninfected tumor cell populations, respectively. Why logistic and not Gompertz, which is generally believed to be more adequate?

**Author response**: *This issue was considered in our previous work. In short, it is quite disputable that *"Gompertz's growth is generally believed to be more adequate". *We chose the logistic law because it is the simplest form whose predictions agree with the empirical data. Moreover, it seems most unlikely that qualitative results change if we use the Gompertz growth law instead of the generalized logistic law*.

(b) The interaction between the two populations is assumed to be governed by the term *βXY*/(*X *+ *Y*). Why is the infection rate assumed proportional to the *relative *size *Y*/(*X *+ *Y*) of the infected population rather than to its absolute size *Y*? Is there any theoretical or experimental rationale for such an assumption?

**Author response**: *This issue was discussed in detail in Ref*. [51].

(c) The assumption that "transmission coefficient *β *is the product of the susceptibility of uninfected cells *β*_1 _and the virus replication rate *β*_2_" (Section *Distributed susceptibility *...) is quite problematic because it contradicts the structure of the model. Imagine the virus replication rate is doubled. However, the term *βXY*/(*X *+ *Y*) would not necessarily double! Therefore, model (11) is also incorrect. The above assumption would be plausible only if the interaction term in the model were *βXY*.

**Author response**: *We would like to clarify that the system (11) is correct if the parameter β*_*2 *_*is attributed to any trait that describes the ability of the virus to infect tumor cells. To justify the use of model (11), we slightly changed the text, in particular, by replacing the term 'virus replication rate' with the more abstract term 'virus virulence' that reflects the general ability of a virus to infect tumor cells. In this setting, the model (11) is correct*.

(d) The formula *E*(*t*) = *E*_*β1 *_(*t*) *E*_*β2 *_(*t*) on p. 17 means that the authors tacitly assumed that parameters *β*_*1 *_and *β*_*2*_, viewed as random variables, are independent. Are they? It seems likely that "virulence" of the virus may affect both virus replication rate *β*_*2 *_and susceptibility of uninfected cells *β*_*1*_.

**Author response**: *Yes, it was assumed that the two parameters are independent. We do not know anything about the dependence between these parameters although it is a natural extension of the model to assume some form of dependence. The independence assumption was chosen to simplify the math. More general situations are also amenable to mathematical analysis given the initial joint distribution (similar to Ref*. [62]*)*.

4. Moment generating function *M*_*P*_(*λ*) of a probability distribution *P *is generally not defined for all real *λ*. This brings about artificial difficulties that are not properly accounted for in the paper. Also, why not to deal with the characteristic function instead and avoid these difficulties altogether?

**Author response**: *Importantly, the current parameter distribution is determined through the mgf of the initial distribution, and not through the characteristic function. Indeed, a mgf cannot be defined for all real λ, but this does not amount to "artificial difficulties". We would like to emphasize that, in some cases, the non-existence of the mgf reflects intrinsic and important problems. In brief, in the MA, it is shown that heterogeneous model (A.2) is equivalent to the Cauchy problem (A.4)–(A.5) if the latter has a unique global solution in *[*0, T*)*. Obviously, this solution does not exist if the corresponding mgf M*(*λ*) *does not exist for some λ = q*(*t*)*, and, consequently, solution of the heterogeneous model does not exist at this t (e.g., there can be a blow-up; for a simple example, see ref*. [2]*in the MA). This is now mentioned in the text (section on *Distributed susceptibility).

5. The authors should draw a careful distinction between mathematical results (in which case they should be proved) and the results of their simulation studies.

**Author response**: *All new mathematical results that are used in the text are now proved in the MA*.

6. Bifurcation analysis and phase portraits of the homogeneous model are too sketchy for their validity to be evaluated. The authors should describe the types *I-VIII *of system behavior in more detail. Are all of them observed in reality? Also, what happens in the case *γ *= 1?

**Author response**: *Detailed bifurcation analysis of the homogeneous model is presented in our recent publication (Ref*. [51]*), so we did not perceive it necessary to reiterate the descriptions here*.

7. How were parameters for the numerical simulations selected? Are they representative of the outcomes?

**Author response**: *The parameters for the numerical simulations were selected such as to illustrate new dynamical regimes of heterogeneous models which are unobservable in homogeneous settings. The parameters used are representative in the sense that a set of close parameter values yields qualitatively similar results*.

8. The content of the appendix provides some mathematical background for the main text but does not seem to support it in more specific ways. In particular, asymptotic analysis that was discussed in the main text was not carried out in the appendix.

**Author response**: *The corresponding changes were made in the main text (section on Distributed susceptibility) and in the Mathematical Appendix)*

### Reviewer's report 2

Natalia Komarova, Department of Mathematics, University of California-Irvine (nominated by Orly Alter)

The paper by Karev et al "Mathematical modeling of tumor therapy with oncolytic viruses: Effects of parametric heterogeneity on cell dynamics" creates a mathematical framework for studying dynamical systems with distributed parameters. This is used to model treatment regimes of cancer with oncolytic viruses. There, tumor heterogeneity is shown to play an important role. It is demonstrated that, depending on the distribution width of the parameters, very complex behaviors can be observed, including tumor dormancy, tumor recurrence and a reversal of treatment success.

I believe that developing a systematic modeling framework for systems with continuously distributed parameters is very important. I therefore recommend the paper for publication. However, I would like the authors to clarify the following points.

(1) The authors emphasized the importance of the variance of the initial distribution of the heterogeneous parameter. I believe that in this model, there is a characteristic of the initial distribution which is even more important than that. It is the support of the initial distribution of the parameter. Simply speaking, since there are no mutations in the model, knowing the types present in the system initially reveals with certainty what will happen in the end.

In fact, the behavior of the system with a distributed parameter can be predicted qualitatively from the following two pieces of information: (i) the direction of selection and (ii) the support of the initial distribution function. For instance, if the transmission coefficient, beta, exhibits heterogeneity, then we know that (i) cells with a lower beta values will be selected for and (ii) the lowest beta from the initial distribution is given by η. Therefore, we know that eventually the cells with *β *= η will outcompete the rest, and the dynamics of the system can thus be predicted.

**Author response**: *We agree that the support of initial distribution is an equally important characteristic of the system. While the parameters of the initial distribution together with system dynamics determine the speed with which the final state is reached, the support determines, in the models considered in the text, the final outcome. We added some text to clarify this point*.

*In general, the assertion that the behavior of the system with a distributed parameter can be predicted given that the direction of selection and the support are known is not valid (although it is true for our particular models). The simplifying fact in our presentation was that the mean parameter values are monotone functions (i.e., we can unambiguously identify the direction of selection). In general, these functions depend on the system dynamics and can be arbitrary (e.g., it is possible to construct a system where they are periodic (Ref*. [65]*))*.

(2) Intuitively speaking, the extreme values of the distributed parameter (e.g. η in the gamma-distribution used by the authors) must be responsible for the final outcome of the dynamics, and the width of the distribution should correlate with the speed with which the system attains its final homogeneous state. Is this true? Is this possible to demonstrate?

**Author response**: *The final outcome in our models depends, as correctly stated in Komarova's remark (1), on the direction of selection and the extreme values of the distributed parameters. E.g., if we have a gamma-distribution on *[η, ∞)*, then the value of η determines the final outcome if individuals with smaller parameter values are more fit, but is not essential for the asymptotic system dynamics in the opposite case*.

*We do not have an analytical solution to the question on correlation between the speed of movement in the parameter space and the width of the distribution. It is generally true, however, that the speed with which the system attains its final state depends on the width of the distribution and on the current parameter values, and the sizes of the cell populations*.

(3) The elegant model presented by the authors deals with continuously distributed parameters. In reality, many parameters can only attain a discrete (and small) set of values. It would be very nice to see (and easy to show) how the mathematical analysis should be modified to deal with discrete distributions. Some of the consequences of the analysis may look more intuitive in this case.

**Author response**: *We explicitly indicate in the revised text that the mathematical framework can equally be applied to continuous or discrete distributions. The mathematical analysis does not have be modified (discrete distributions were used, e.g., in (Karev, 2003, Ecol. Model. 160, 23–37). For example, if we assume that the initial distribution of the parameter β that describes susceptibility of uninfected tumor cells is Poissonian with initial mean β*_0 _*then, using mgf for the Poisson distribution, we obtain E*_*β *_(*t*) = *β*_0 _exp(*q*(*t*))*. The latter expression can be used in system (5)–(6)*.

(4) I found the mathematical derivation presented in the appendix a little hard to read. It would help if the authors showed that the distribution *x*(*t*, *β*) (normalized) is equal to the distribution exp(*β q*) *p *(0, *β*) (normalized) if the variables *x *and *q *satisfy the given equations.

**Author response**: *The proof of Theorem 1 contained some typos which might have led to confusion; this was corrected [see *Additional file 1*]*.

### Reviewer's report 3

David Krakauer, Santa Fe Institute

In this paper Karev et al extend their earlier work on the dynamical properties of an implicit oncolytic dynamic (virus is not treated and assumed through a separation of time scales to be stationary) to include continuous variation in the viral traits transmission coefficients and cytotoxicity. The model comprises a system of ODEs tracking mean densities of uninfected and infected cells including time varying mean transmissibility and cytotoxicity.

In this more complex model with heterogeneity, the range of dynamical behaviors is expanded, manifesting patterns of tumor recurrence, and quasi-periodic orbits in cell densities. The paper raises interesting questions about the possibility of systematic, positive intervention into such a complex system.

While the distributional approach of this paper is timely and expands the range of model behaviors, it remains a continuous, deterministic treatment and the stochastic implication of rare events stemming from the tails of distributions can not be understood. And I would hypothesize that it is these improbable events that are more typically associated with recurrence expanding from small populations of concealed cells.

**Author response**: *Indeed, stochastic implications of rare random events cannot be understood within the framework of this paper because the models are deterministic. We use a probabilistic distribution to describe the parametric heterogeneity of tumor cells. The tails of distributions considered in the paper show only that there are subpopulations of cells with relatively high or low parameter values, and the proportion of these subpopulations is small. We agree that "improbable events" implicated by Krakauer are important in carcinogenesis, and in cancer recurrence in particular. However, we would like to stress that our model shows that not only such events can lead to, e.g., tumor recurrence, but also existence of small populations of cells whose probability to be infected by an oncolytic virus is relatively small but still essentially non-zero*.

A general problem I have with the paper, that will probably diminish its impact, is that no clearly important insights are generated by the model, but rather a range of complex behaviors which probably need further analysis. Since this paper offers qualitative results, I view this as a failing, as the purpose of such models is largely to sharpen intuition. One problem throughout is identifying clearly the source of heterogeneity – is this of viral or cellular origin? In the model this remains ambiguous.

**Author response**: *Obviously, most biological systems are heterogeneous including the population of tumor cells. The whole point of this paper is whether a heterogeneous model allows to describe qualitatively new phenomena in comparison to the corresponding homogeneous model. The models described here possess, e.g., regimes of i) tumor recurrence, ii) transiently constant tumor size, iii) quasi-chaotic behavior. Thus, it seems that this work, actually, does sharpen intuition and even yields results that might not be intuitively obvious. Furthermore, we would like to emphasize that we propose a new modeling approach to deal with parametric heterogeneity, which is computationally and, in some cases, analytically feasible, and which can hopefully be applied to other existing models of ODEs for cancer progression and treatment*.

*The source of heterogeneity in our models can be of viral or cellar origin. When describing each specific model, we tried to explain possible sources of heterogeneity. For instance, when we deal with distributed susceptibility, the source is cellular. Viral heterogeneity is included in the models through the infected cell population because we do not treat the virus population explicitly. We agree that explicit virus dynamics would improve and clarify the models, but it also would complicate considerably the full parametric analysis of the homogeneous model*.

I wonder if the authors could not shorten the paper and really focus on its most important insights. I think that these would include: (1) Increased resilience of tumors stemming from heterogeneity, (2) The increased success of oncolytic treatment with heterogeneous virus (if this is a real result of the model), (3) the impact of variance of heterogeneity on dynamics, (4) the impact of the distribution, (5) the clearly stated implications for therapy.

**Author response**: *We believe that all these issues are addressed in the paper; we do not see how the article could be shortened significantly without losing information*.

I also have some specific comments which overlap with those I made in a review of the author's previous paper in this series.

1. I feel that not explicitly treating the virus misses important problems and would make the interpretation of model parameters much clearer.

**Author response**: *As indicated above, we agree but this would make the model substantially less tractable*.

2. I am worried about the requirement that complete information about the distributions needs to be known in order to track the means in the ODEs. I am guessing that not only the shape but the form of the distributions could change over the course of infection.

**Author response**: *Formally, to obtain a solution of the system at any time moment, including t → ∞, we have to know the exact form of initial distributions. It should be noted, however, that, to predict the behavior of the system on relatively short time spans, several moments of the distribution might be enough*.

*The question on the form of distributions can be treated mathematically (for a closely related model, see Ref*. [62]*. The fact is that a number of important distributions maintain their form over time. For example, if the initial distribution is gamma-distribution, it can be rigorously proved that it will always remain a gamma-distribution with parameters changing with time. The same statement is valid for normal distribution and others. However, there are distributions that change their form. For example, a uniform distribution becomes exponential*.

3. The model is strictly speaking ecological as all variants are assumed to be present at *t*_*0*_. My understanding is that cancer progresses through mutational events and these are not treated in this model.

**Author response**: *We agree with this remark. Our model does not consider mutations. In our model, all variants are present at the initial time moment, perhaps, with extremely low densities*.

4. Typically heterogeneity is spatial across a tissue.

**Author response**: *The question on relation of spatial and parametric heterogeneity is important and interesting. However, in this work, we do not consider the spatial structure*.

5. I still feel that a very important aspect of oncolytic therapy is the problem of target-degeneracy leading to infection of non-cancer cells. As these are not treated in this paper I remain suspicious of the efficacy of the complete clearance equilibrium. I think it would be interesting to explicitly include cell heterogeneity in order to treat both cell populations – healthy and cancerous.

**Author response**: *This issue was, actually, addressed in our response to Krakauer's comments on our previous paper (Ref*. [51]*). There are, indeed, many ways to expand these models. We stuck to a minimal version in order to keep the model within analytic solvability*.

## Supplementary Material

Additional file 1 Mathematical appendix. The Mathematical appendix contains proofs of theorems and additional mathematical details. Click here for file
