# Supplementary material for: Mathematical modeling of tumor therapy with oncolytic viruses: effects of parametric heterogeneity on cell dynamics
Source: Biol Direct. 2006 Oct 3;1:30. doi: 10.1186/1745-6150-1-30 (PMC1622743; doi:10.1186/1745-6150-1-30)
Supplement: Additional file 1 — Mathematical appendix. The Mathematical appendix contains proofs of theorems and additional mathematical details. [file 1745-6150-1-30-S1.doc]

**Mathematical Appendix**

**Mathematical modeling of tumor therapy with oncolytic viruses:**

**Effects of parametric heterogeneity on cell dynamics**

Georgy P. Karev^1^ , Artem S. Novozhilov^1^, Eugene V. Koonin^1,*^

^1^National Center for Biotechnology Information, National Library of Medicine, National Institutes of Health, Bethesda, MD 20894, USA.

*To whom correspondence should be addressed. Email [koonin@ncbi.nlm.nih.gov](mailto:koonin@ncbi.nlm.nih.gov)

**On Mathematical Theory of Inhomogeneous Community Models**

Let us consider a model of a community consisting of *m* interacting populations in the general form

*dN j/dt* = *N ^j^F ^j^*(**N***,***a**), *j*=1,…*m*,

where *N ^j^* is the size of *j*-th population, **N*=***(*N*^1^*, …,N ^m^*). The growth rate *F ^j^* can depend on parameters and **a*=***(*a*_1_*,a*_2_*,…a_s_*) is the vector-parameter with the domain ***A***. We suppose here that every individual is characterized by its own value of vector-parameters **a***.* Let *l^j^*(*t,***a**) be the density of the *j*-th population along the vector-parameter **a** at time *t*. In [1-3] the following model of an inhomogeneous community was explored:

$\frac{\partial l^{j}(t,\mathbf{a})}{\partial t}$ = *F ^j^*(**N**,**a**) *l ^j^*(*t,***a**), (A.1)

*N ^j^*(*t*) = *l ^j^*(*t,****a***)d***a*** *.*

We suppose here that the fitness of individuals in the *j*-th population, defined by the formula *F^j^*(**N**,**a**)=[d*l ^j^*(*t,***a**)/d*t*]/*l ^j^*(*t,***a**), depends on the parameter **a** and on total sizes of all subpopulations but not on the parameter distribution inside the subpopulations.

This type of models can well describe, e. g., cooperative dynamics of populations or resource-consumer model of inhomogeneous community, but are not applicable to prey-predator or epidemiological problems, where the interactions between populations should be “balanced” (e.g., the number of newly infected individuals should be equal to decrease of the number of susceptible individuals). Nevertheless, the main ideas and techniques used in [1] can be applied to “balanced” inhomogeneous community models with slight modifications.

We do not develop here a general theory of such models; instead, we consider a model of a biological community composed of two interacting populations depending on parameters:

$\begin{matrix} \frac{\partial x(t,a)}{\partial t}=x(t,a)[F_{1}(X,Y,E_{b}(t))+aG_{1}(X,Y,E_{b}(t))], \\ \frac{\partial y(t,b)}{\partial t}=y(t,b)[F_{2}(X,Y,E_{a}(t))+bG_{2}(X,Y,E_{a}(t))], \end{matrix}$ (A.2)

assuming that parameters *a* and *b* are distributed. Here $x(t,a)$, $y(t,b)$ are the population densities, $X(t)=\int_{A} x(t,a)da$, $Y(t)=\int_{B} y(t,b)db$) are the total sizes of the cell populations. Denote *p*_1_(*t,a*)=*x*(*t,a*)/*X*(*t*), *p*_2_(*t,b*)=*y*(*t,b*)/*Y*(*t*) the current probability density functions of corresponding parameters in uninfected and infected populations accordingly; then

$E_{a}(t)=\left(\int_{A} ax(t,a)da\right)/X(t)=\int_{A} ap_{1}(t,a)da$, $E_{b}(t)=\left(\int_{B} by(t,b)db\right)/Y(t)=\int_{B} bp_{2}(t,b)db$

are the current mean values of the parameters *a* and *b* .

We suppose that the initial population sizes and initial distributions of the parameters are given:

$x(0,a)=X_{0}p_{1}(0,a),\text{ }y(0,b)=Y_{0}p_{2}(0,b)$.

The particular form of system (A.2) is motivated by the models explored in the paper, which are special cases of (A.2).

Problem (A.2) can be reduced to the Cauchy problem for a certain system of ODE. Let

*M_a_*(λ)=exp(*λa*)*p*_1_(0, *a*)d*a*

*M_b_*(λ)=exp(λ*b*)*p*_2_(0, *b* )d*b,* (A.3)

be the mgfs (moment generating functions) of the initial probability densities *p*_1_(0,*a*) and *p*_2_(0,*b*).

Let us consider the system for auxiliary variables

d*q*^1^_0_(*t*)/d*t= q*^1^_0_(*t*)*F*_1_(*X*,Y*, E*_1_(*t*)), *q*^1^_1_(0)=1; (A.4)

d*q*^1^_1_(*t*)/d*t= G*_1_(*X*,Y*, E*_1_(*t*)), *q*^1^_1_(0)=0;

d*q*^2^_0_(*t*)/d*t= q*^2^_0_(*t*) *F*_2_(*X*,Y*, E*_2_(*t*)), *q*^2^_0_(0)=1;

d*q*^2^_1_(*t*)/d*t= G*_2_(*X*,Y*, E*_2_(*t*)), *q*^2^_1_(0)=0.

The functions *X**(*t*)*, Y**(*t*) and *E_i_*(*t*) are defined by the formulas

$E_{1}(t)=\left.\frac{1}{M_{a}(q^{1}_{1}(t))}\frac{dM_{a}(\lambda)}{d\lambda}\right|_{\lambda=q^{1}_{1}(t)},\text{ }E_{2}(t)=\left.\frac{1}{M_{b}(q^{2}_{1}(t))}\frac{dM_{b}(\lambda)}{d\lambda}\right|_{\lambda=q^{2}_{1}(t)}$ (A.5)

*X**(*t*)= *X*(0) *q*^1^_0_(*t*) *M_a_*(*q*^1^_1_(*t*)), *Y**(*t*)= *Y*(0) *q*^2^_0_(*t*) *M_b_*(*q*^2^_1_(*t*)).

**Theorem 1.** *Let Cauchy problem* (A.4)-(A.5) *have a unique global solution* {*q ^j^_i_*(*t*)} *at t*∈[0*,T*) *where* 0≤*T*≤∞. *Then the functions*

*x*(*t,a*) = *x*(0*,a*) *q*^1^_0_(*t*) exp[*a q*^1^_1_(*t*)], (A.6)

*y*(*t,b*) = *y*(0*,b*) *q*^2^_0_(*t*) exp[*b q*^2^_1_(*t*)],

*X*(*t*) = *X*(0) *q*^1^_0_(*t*) *M_a_*(*q*^1^_1_(*t*)),

*Y*(*t*) = *Y*(0) *q*^2^_0_(*t*) *M_b_*(*q*^2^_1_(*t*))

*satisfy system* (A.2) *at t*∈[0*,T*)*.*

*Conversely, if x*(*t,a*), *y*(*t,b*), *and X*(*t*)*= x*(*t,a*)d*a, Y*(*t*) *=y*(*t,b*)d*b satisfy system* (A.2) *at t*∈[0*,T*)*, then Cauchy problem* (A.4) *has a global solution* {*q ^j^_i_* (*t*)} *at t*∈[0*,T*) *and these functions can be written in the form* (A.6) *at t*∈[0*,T*)*.*

Proof.

Let {*q^i^_j_*(*t*)} be a solution of Cauchy problem (A.4)-(A.5) at *t*∈[0,*T*). According to system (A.4),

$\frac{\partial x(t,a)}{\partial t}$*= x*(0*, a*) exp[*a q*^1^_1_(*t*)] {d*q*^1^_0_(*t*)/d*t + q*^1^_0_(*t*)*a* d*q*^1^_1_(*t*)/d*t*} =

*x*(*t,a*){*F*_1_(*X*,Y*, E*_1_(*t*)) + *a G*_1_(*X*,Y*, E*_1_(*t*))] ;

$\frac{\partial y(t,b)}{\partial t}$*= y*(0*, b*) exp[*bq*^2^_1_(*t*)] {d*q*^2^_0_(*t*)/d*t + q*^2^_0_(*t*)*b*d*q*^2^_1_(*t*)/d*t*} =

*y*(*t,b*){*F*_2_(*X*,Y*, E*_2_(*t*)) + *bG*_2_(*X*,Y*, E*_2_(*t*))].

Next,

*x*(*t,a*)d*a=x*(0*,a*) *q*^1^_0_(*t*) exp[*a q*^1^_1_(*t*)] d*a=X*(0) *q*^1^_0_(*t*) *M_a_*(*q*^1^_1_(*t*));

*y*(*t,b*)d*b=y*(0*,b*) *q*^2^_0_(*t*)exp[*b q*^2^_1_(*t*)] d*b =Y*(0) *q*^2^_0_(*t*)*M_b_*(*q*^2^_1_(*t*)).

Hence, the functions defined by (A.6), are connected by the relations

*X*(*t*)= *x*(*t,a*)d*a*, *Y*(*t*)= *y*(*t;b*)d*b.*

To complete the proof of the first part of the theorem, we have to determine the current parameter distributions, $p_{1}(t,a)$ and $p_{2}(t,b)$, and show that *E_i_*(*t*) defined by (A.5) are equal to the current mean values of the parameters, i.e., *E_a_*(*t*)=$\int_{A} ap_{1}(t,a)da$=*E*_1_(*t*) and *E_b_*(*t*)=$\int_{B} bp_{2}(t,b)db$ =*E*_2_(*t*). For this, we need the following

**Proposition 1**. *Let the Cauchy problem* (A.4)-(A.5) *have a unique solution* {*q^j^_i_*(*t*)} *at t*∈[0*,T*). *Then, for all* *t*∈[0*,T*),

1) *the current parameter distributions,* $p_{1}(t,a)$ and $p_{2}(t,b)$ *are equal to*

$p_{1}(t,a)$=$p_{1}(0,a)$exp[*a q*^1^_1_(*t*)] / *M_a_*(*q*^1^_1_(*t*)), (A.7)

$p_{2}(t,b)$=$p_{2}(0,b)$exp[*b q*^2^_1_(*t*)] / *M_b_*(*q*^2^_1_(*t*));

2) *the moment generation functions of* $p_{1}(t,a)$ and $p_{2}(t,b)$ *are equal to*

*M_a_*(*t*; λ)= *M_a_*(λ*+q*^1^_1_(*t*)) / *M_a_*(*q*^1^_1_(*t*))*,* (A.8)

*M_b_*(*t*; λ)= *M_b_*(λ*+q*^2^_1_(*t*)) / *M_b_*(*q*^2^_1_(*t*))

*where M_a_*(λ), *M_b_*(*λ*) *are the mgf of initial distributions* (A.3).

Proof.

By definition, $p_{1}(t,a)$ = *x*(*t,a*)/*X*(*t*), $p_{2}(t,b)$ = *y*(*t, b*)/*Y*(*t*). According to (A.6),

$p_{1}(t,a)$= *x*(0*,a*) *q*^1^_0_(*t*) exp[*a q*^1^_1_(*t*)] / *X*(*t*).

Further, *X*(*t*) = *X*(0) *q*^1^_0_(*t*) *M_a_*(*q*^1^_1_(*t*)) by (A.5), so

$p_{1}(t,a)$= $p_{1}(0,a)$exp[*a q*^1^_1_(*t*)] / *M_a_*(*q*^1^_1_(*t*)).

Similarly, $p_{2}(t,b)$=$p_{2}(0,b)$exp[*b q*^2^_1_(*t*)] / *M_b_*(*q*^2^_1_(*t*)). Next,

*M_a_*(*t*; λ)= exp(λ*a*)$p_{1}(t,a)$d*a=* {exp[(λ+*q*^1^_1_(*t*))*a*] $p_{1}(0,a)$d*a*}/ *M_a_*(*q*^1^_1_(*t*)) *=*

*M_a_*(λ*+q*^1^_1_(*t*)) / *M_a_*(*q*^1^_1_(*t*)).

Similarly, *M_b_*(*t*; λ)= *M_b_*(λ*+q*^2^_1_(*t*)) / *M_b_*(*q*^2^_1_(*t*)), as desired.

Now we are able to complete the proof of part 1 of Theorem:

*E_a_*(*t*)= $\int_{A} ap_{1}(t,a)da$=∂ *M_a_*(*t*; λ) /∂*λ*│*_λ=_*_0_=[∂*M_a_*(λ*+q*^1^_1_(*t*)) /∂*λ*│*_λ=_*_0_] / *M_a_*(*q*^1^_1_(*t*)),

and the last expression coincides with the formula (A.5) for *E*_1_(*t*).

Similarly, *E_b_*(*t*)= ∂ *M_b_*(*t*; λ) /∂*λ*│*_λ=_*_0_=[∂*M_b_*(λ*+q*^2^_1_(*t*)) /∂*λ*│*_λ=_*_0_] / *M_b_*(*q*^2^_1_(*t*)).

Let us prove now part 2 of Theorem; let *x*(*t, a*), *y*(*t,b*), and *X*(*t*)*= x*(*t,a*)d*a, Y*(*t*) *= y*(*t*,*b*)d*b* satisfy system (A.2) at *t*∈[0*,T*), so that

$$\begin{matrix} \frac{\partial x(t,a)}{\partial t}=x(t,a)[F_{1}(X,Y,E_{b}(t))+aG_{1}(X,Y,E_{b}(t))], \\ \frac{\partial y(t,b)}{\partial t}=y(t,b)[F_{2}(X,Y,E_{a}(t))+bG_{2}(X,Y,E_{a}(t))], \end{matrix}$$

where

$E_{a}(t)=\left(\int_{A} ax(t,a)da\right)/X(t)$, $E_{b}(t)=\left(\int_{B} by(t,b)db\right)/Y(t)$*.*

Let us temporarily define the functions

*q*^1^_0_(*t*) *=* exp[*F*_1_(*X*(*s*)*,Y*(*s*), *E_b_*(*s*)) d*s*]*, q*^1^_1_(*t*) *=G*_1_(*X*(*s*)*,Y*(*s*), *E_b_*(*s*)) d*s,*

*q*^2^_0_(*t*) *=* exp[*F*_2_(*X*(*s*)*,Y*(*s*), *E_a_*(*s*)) d*s*]*, q*^2^_1_(*t*) *=G*_2_(*X*(*s*)*,Y*(*s*), *E_a_*(*s*)) d*s.*

Then

$\frac{\partial x(t,a)}{\partial t}\frac{1}{x(t,a)}$ = $\frac{dq_{0}^{1}(t)}{dt}\frac{1}{q_{0}^{1}(t)}$ + *a*$\frac{dq_{1}^{1}(t)}{dt}$,

$\frac{\partial y(t,b)}{\partial t}\frac{1}{y(t,b)}$ = $\frac{dq_{0}^{2}(t)}{dt}\frac{1}{q_{0}^{2}(t)}$ + *b*$\frac{dq_{1}^{2}(t)}{dt}$,

hence

ln *x*(*t,a*) = ln*q*^1^_0_(*t*) + *aq*^1^_1_(*t*) +*C*^1^

ln *y*(*t,b*) = ln *q*^2^_0_(*t*) + *bq*^2^_1_(*t*) + *C*^2^

where *C ^j^* do not depend on *t*.

Taking *C*^1^ *=*ln *x*(0*,a*), *C*^2^ *=*ln *y*(0*,b*) we obtain that

*x*(*t,a*) = *x*(0*,a*) *q*^1^_0_(*t*) exp[*aq*^1^_1_(*t*)],

*y*(*t,b*) = *y*(0*,b*) *q*^2^_0_(*t*) exp[*bq*^2^_1_(*t*)].

Hence

*X*(*t*)≡ *x*(*t,a*)d*a=x*(0*,a*) *q*^1^_0_(*t*) exp[*aq*^1^_1_(*t*)] d*a=X*(0) *q*^1^_0_(*t*) *M_a_*(*q*^1^_1_(*t*)),

*Y*(*t*)≡ *y*(*t,b*)d*b= y*(0,*b*) *q*^2^_0_(*t*) exp[*bq*^2^_1_(*t*)]d*b* =*Y*(0) *q*^2^_0_(*t*) *M_b_*(*p*^2^_1_(*t*)) at *t*∈[0,*T*).

From the definition, {*q^i^_j_*(*t*)} is a solution of Cauchy problem (A.4)-(A.5) for *t*∈[0,*T*).

The proof is completed.

**Corollary 1**. System (A.2) is equivalent to the system (A.7) for the current parameter distributions and the following non-autonomous system of ODEs for the total population sizes:

$\begin{matrix} \frac{dX}{dt}=X[F_{1}(X,Y,E_{b}(t))+E_{a}(t)G_{1}(X,Y,E_{b}(t))], \\ \frac{dY}{dt}=Y[F_{2}(X,Y,E_{a}(t))+E_{b}(t)G_{2}(X,Y,E_{a}(t))], \\ X(0)=X_{0},Y(0)=Y_{0} \end{matrix}$ (A.9)

where the mean parameter values are

$E_{a}(t)=\left.\frac{1}{M_{a}(q_{1}(t))}\frac{dM_{a}(\lambda)}{d\lambda}\right|_{\lambda=q_{1}(t)},\text{ }E_{b}(t)=\left.\frac{1}{M_{b}(q_{2}(t))}\frac{dM_{b}(\lambda)}{d\lambda}\right|_{\lambda=q_{2}(t)}$ (A.10)

and *M_a_*(*λ*) and *M_b_*(*λ*) are the mgf of the given initial pdfs$p_{1}(0,a)\text{ and }p_{2}(0,b)$. The auxiliary variables *q*_1_(*t*), *q*_2_(*t*) can be found from the system

$\frac{dq_{1}}{dt}=G_{1}(X,Y,E_{a}(t)),\text{ }\frac{dq_{2}}{dt}=G_{2}(X,Y,E_{b}(t)),\text{ }q_{1}(0)=0,\text{ }q_{2}(0)=0$. (A.11)

Proof.

It is enough to prove that system (A.7), (A.9)-(A.11) is equivalent to (A.4)-(A.5).

Let Cauchy problem (A.4)-(A.5) have a unique global solution {*q ^j^_i_*(*t*)} at *t*∈[0*,T*). Then, according to Theorem 1, functions (A.6) solve (A.2); it is easy to check that *X*(*t*) = *X*(0) *q*^1^_0_(*t*) *M_a_*(*q*^1^_1_(*t*)), *Y*(*t*) = *Y*(0) *q*^2^_0_(*t*) *M_b_*(*q*^2^_1_(*t*)) solve (A.9) and the definitions of the auxiliary variables and mean values of the parameters coincide with (A.10)-(A.11). Next, if the current parameter distributions $p_{1}(t,a)$, $p_{2}(t,b)$ are defined by (A.7), then $x(t,a)=X(t)p_{1}(t,a),$ $y(t,b)=Y(t)p_{2}(t,b)$solve (A.2).

Conversely, let *X*(*t*), *Y*(*t*), *q*_1_(*t*), *q*_2_(*t*) solve (A.9)-(A.11) at *t*∈[0*,T*). Let *q*^1^_1_(*t*) *= q*_1_(*t*),  *q*^2^_1_(*t*) *= q*_2_(*t*) and

*q*^1^_0_(*t*)*=*exp[*F*_1_(*X*(*s*)*,Y*(*s*),*E_b_*(*s*))d*s*]*, q*^2^_0_(*t*)*=*exp[*F*_2_(*X*(*s*)*,Y*(*s*),*E_a_*(*s*))d*s*].

Then {*q^j^_i_*(*t*)} is a solution of Cauchy problem (A.4)-(A.5); according to Proposition 1, the mgfs of the current distributions are given by (A.8) and mean parameter values (A.10) coincide with that defined by formulas (A.5). Q.E.D.

**Corollary 2.** *The current mean parameter values that are determined by formulas (A.5) satisfy the equations*

$\frac{dE_{a}(t)}{dt}=\sigma_{a}^{2}(t)G_{1}(X,Y,E_{b}(t)),\text{ }\frac{dE_{b}(t)}{dt}=\sigma_{b}^{2}(t)G_{2`}(X,Y,E_{a}(t))$*,* (A.12)

*where $\sigma_{a}^{2}(t)\geq0$ and $\sigma_{b}^{2}(t)\geq0$ are the current variances of the parameter distributions.*

Proof.

Differentiating (A.5), using (A.8), (A.11), and the fact that $\sigma^{2}(t)=\left.\frac{d^{2}M(t,\lambda)}{d\lambda^{2}}\right|_{\lambda=0}-E^{2}(t)$, we obtain the necessary equations. Q.E.D.

**Example 1**. Let parameter *a* have the Γ-distribution at the initial instant with coefficients *s, η, k*:

*P*(0,*a*)= *s^k^* (*a* - *η*)*^k^*^-1^exp[-(*a* - *η*)*s*]/*Γ*(*k*),

with the mean *μ*= *η* + *k /s,* the variance *σ* ^2^ = *k/s*^2^ and the mgf *M_a_*(*λ*) = exp(λ*η*) /(1-λ/*s*) *^k^.* Then

*M_a_*(*t*; λ)= *M_a_*(λ*+q*_1_(*t*)) / *M_a_*(*q*_1_(*t*))= exp(λ*η*) /(1-λ/(*s- q*_1_(*t*)) *^k^* ,

i.e., the parameter *a* has again the Γ-distribution with coefficients *s-q*_1_(*t*)*, η, k* at any moment *t<T* such that the solution *q*_1_(*t*)< *s* exists for $t\in[0,T)$*.* Next,

∂*M* (*λ+q*_1_(*t*)) /∂*λ*│*_λ=_*_0_ / *M*(*q*_1_(*t*))=*k*/(*s*-*q*_1_(*t*))+*η.*

Hence, the current mean of the parameter is

$E_{a}(t)=\frac{k}{s-q_{1}(t)}+\eta$ (A.13)

where the auxiliary variable *q*_1_(*t*) is defined by (A.11).

As a particular inhomogeneous community model let us consider system (7):

$\begin{matrix} \frac{\partial x(t,\beta)}{\partial t}=x(t,\beta)\left[1-(X(t)+Y(t))\right]-\frac{\beta x(t,\beta)Y(t)}{X(t)+Y(t)}, \\ \frac{\partial y(t,\delta)}{\partial t}=\gamma y(t,\delta)\left[1-(X(t)+Y(t))\right]+\frac{E_{\beta}(t)X(t)y(t,\delta)}{X(t)+Y(t)}-\delta y(t,\delta). \end{matrix}$ (A.14)

Suppose that, at the initial moment, both parameters β and δ have the Γ-distribution with coefficients *s*_1_*, η*_1_*, k*_1_ and *s*_2_*, η*_2_*, k*_2_, respectively. Then the current mean parameter values are given by (A.13) and, finally, system (A.14) is reduced to the following system of ODEs:

$$\frac{dq_{1}}{dt}=-\frac{Y}{X+Y},\text{ }\frac{dq_{2}}{dt}=-1,\text{ }$$

$$\begin{matrix} \frac{dX}{dt}=X(1-(X+Y))-(\frac{k_{1}}{s_{1}-q_{1}(t)}+\eta_{1})\frac{XY}{X+Y}, \\ \frac{dY}{dt}=\gamma Y(1-(X+Y))+(\frac{k_{1}}{s_{1}-q_{1}(t)}+\eta_{1})\frac{XY}{X+Y}-(\frac{k_{2}}{s_{2}-q_{2}(t)}+\eta_{2})Y, \\ X(0)=X_{0},Y(0)=Y_{0},\text{ }q_{1}(0)=0,\text{ }q_{2}(0)=0. \end{matrix}$$

References:

1. Karev GP: Heterogeneity effects in population dynamics. *Doklady Mathematics* 2000, 62(1):141-144.

2. Karev GP: Dynamics of inhomogeneous populations and global demography models. *Journal of Biological Systems* 2005, 13(1):83-104.

3. Novozhilov AS: Analysis of a generalized population predator-prey model with a parameter distributed normally over the individuals in the predator population. *Journal of Computer and Systems Sciences International* 2004, 43(3):378-382.
